# The *Entamoeba histolytica* TBP and TRF1 transcription factors are GAAC-box binding proteins, which display differential gene expression under different stress stimuli and during the interaction with mammalian cells

**DOI:** 10.1186/s13071-018-2698-7

**Published:** 2018-03-07

**Authors:** Ravi Kumar Narayanasamy, Carlos Alberto Castañón-Sanchez, Juan Pedro Luna-Arias, Guillermina García-Rivera, Bartolo Avendaño-Borromeo, María Luisa Labra-Barrios, Jesús Valdés, María Esther Herrera-Aguirre, Esther Orozco

**Affiliations:** 1 Departamento de Infectómica y Patogénesis Molecular, Centro de Investigación y de Estudios Avanzados del Instituto Politécnico Nacional (Cinvestav-IPN), Av. Instituto Politécnico Nacional 2508, Col. San Pedro Zacatenco, C.P, 07360 Ciudad de México, Mexico; 2 Programa de Biomedicina Molecular, Escuela Nacional de Medicina y Homeopatía del Instituto Politécnico Nacional (ENMH-IPN), Guillermo Massieu Helguera 239, Col. La Escalera, C.P, 07320 Ciudad de México, Mexico; 3 Laboratorio de Investigación Biomédica, Subdirección de Enseñanza e Investigación, Hospital Regional de Alta Especialidad de Oaxaca, Aldama S/N, San Bartolo Coyotepec, C.P, 71256 Oaxaca, Mexico; 4 Departamento de Biología Celular, Centro de Investigación y de Estudios Avanzados del Instituto Politécnico Nacional (Cinvestav-IPN), Av. Instituto Politécnico Nacional 2508, Col. San Pedro Zacatenco, C.P, 07360 Ciudad de México, Mexico; 5 Departamento de Bioquímica, Centro de Investigación y de Estudios Avanzados del Instituto Politécnico Nacional (Cinvestav-IPN), Av. Instituto Politécnico Nacional 2508, Col. San Pedro Zacatenco, C.P, 07360 Ciudad de México, Mexico

**Keywords:** *Entamoeba histolytica*, EhTBP, EhTRF1, GAAC-box mutation, Different stress stimuli, Gene knockdown, Differential gene expression

## Abstract

**Background:**

*Entamoeba histolytica* is the protozoan parasite responsible for human amebiasis. It causes up to 100,000 deaths worldwide each year. This parasite has two closely related basal transcription factors, the TATA-box binding protein (EhTBP) and the TBP-related factor 1 (EhTRF1). TBP binds to the canonical TATTTAAA-box, as well as to different TATA variants. TRF1 also binds to the TATTTAAA-box. However, their binding capacity to diverse core promoter elements, including the GAAC-element, and their role in gene regulation in this parasite remains unknown.

**Methods:**

EMSA experiments were performed to determine the binding capacity of recombinant TBP and TRF1 to TATA variants, GAAC and GAAC-like boxes. For the functional analysis under different stress stimuli (e.g. growth curve, serum depletion, heat-shock, and UV-irradiation) and during the interaction with mammalian cells (erythrocytes, MDCK cell monolayers, and hepatocytes of hamsters), RT-qPCR, and gene knockdown were performed.

**Results:**

Both transcription factors bound to the different TATA variants tested, as well as to the GAAC-boxes, suggesting that they are GAAC-box-binding proteins. The *K*_*D*_ values determined for TBP and TRF1 for the different TATA variants and GAAC-box were in the range of 10^-12^ M to 10^-11^ M. During the death phase of growth or in serum depletion, *Ehtbp* mRNA levels significantly increased, whereas the mRNA level of *Ehtrf*1 did not change under these conditions. *Ehtrf*1 gene expression was negatively regulated by UV-irradiation and heat-shock stress, with no changes in *Ehtbp* gene expression. Moreover, *Ehtrf*1 gene also showed a negative regulation during erythrophagocytosis, liver abscess formation, and a transient expression level increase at the initial phase of MDCK cell destruction. Finally, the *Ehtbp* gene knockdown displayed a drastic decrease in the efficiency of erythrophagocytosis in G3 trophozoites.

**Conclusions:**

To our knowledge, this study reveals that these basal transcription factors are able to bind multiple core promoter elements. However, their immediate change in gene expression level in response to different stimuli, as well as during the interaction with mammalian cells, and the diminishing of erythrophagocytosis by silencing the *Ehtbp* gene indicate the different physiological roles of these transcription factors in *E. histolytica*.

**Electronic supplementary material:**

The online version of this article (10.1186/s13071-018-2698-7) contains supplementary material, which is available to authorized users.

## Background

*Entamoeba histolytica* is the causative agent of human amebiasis leading to 100,000 deaths per year [1]. The *Entamoeba histolytica* genome possesses 8201 protein-coding genes, including virulence genes and canonical transcription factors related to the basal transcription machinery [2, 3]. The characterization of some gene promoters, such as those of the *hgl5*, *fdx*, *Ehpgp1, Ehpgp5* and *actin* genes [4–8] has allowed the identification of key elements in promoters, including the TATA-box (GTATTTAAAG/C), Inr (AAAAATTCA), GAAC (AATGAACT) and GAAC-like (GAACTACAAA) core promoter elements, as well as other motifs, including the H_2_O_2_-regulatory motif (HRM) [9], URE-1 [10, 11], URE-3 [12], URE-4 [13], CCAAT-box [14], and CCCCCC motif [15]. The unusual core promoter element GAAC-box is described as a key player in driving the transcription start site of the *hgl5* gene promoter lacking both the TATA and Inr-boxes [4]. An *in silico* analysis of promoters for 246 genes showed that 43 genes contained the GAAC-box, 29 genes with the TATA-box, and 7 genes with both core elements [16]. In addition, it was found that 56% of 4000 genes contain the GAAC-like box [17]. Thus, the genome of this unicellular parasite is equipped with diverse promoter elements that coordinate transcriptional regulation. Several transcription factors have been identified and characterized in this parasite, including the TATA-box binding protein (EhTBP) [18, 19], EhEBP1, EhEBP2, EhHRM-BP, EhURE-BP, EhMyb, STAT, GATA, HSF [20], and the p53-like protein [21]. Recently, EhPC4 was identified as a key player affecting ploidy and genome stability in *E. histolytica* [22].

Multicellular organisms have evolved proteins related to the TATA-box binding protein (TBP) known as TBP-related factors (TRFs) for specialized functions. TRF1 was first described in *Drosophila melanogaster* as involved in the regulation of specific gene subsets in the central nervous system and germ cells [23]. TRF2 has been broadly identified in metazoans, including *Homo sapiens* [24]. The deletion of TRF2 was lethal for embryos of *Caernorhabditis elegans* and *D. melanogaster* [25, 26]. TRF2 selectively regulates TATA-less promoters by driving transcription of essential genes for chromosomal organization, as well as the *PCNA* and *Tudor* genes of *Drosophila* [26]. TRF3 is specific to vertebrates and is widely expressed in various human tissues and cell lines [27]. In the case of unicellular organisms, *Crypthecodinium cohnii* has a particular TBP, which is an intermediate class of transcription factor between TBP and TRFs [28], while *Trypanosoma brucei* has TRF4, which is divergent from metazoan TBPs and is considered as the universal regulator of transcription in this parasite because it is involved in transcription by the three RNA polymerases [29].

*Entamoeba histolytica* has two *tbp* genes, *Ehtbp1* and *Ehtbp2*, which encode proteins of 234 and 212 amino acids, respectively. EhTBP2 is 100% identical to amino acids 23–234 of EhTBP1 and is endogenously silenced [18, 30, 31]. *Entamoeba histolytica* also contains a TBP-related factor, named EhTRF1, which has a highly conserved C-terminal domain with 42.6% identity and 73.7% similarity to the full-length EhTBP. Like other members of the TBP family, both proteins possess a saddle-like structure [30]. EhTBP has the capacity to bind both the TATA-box and different TATA-box variants in vitro, with dissociation constants (*K*_*D*_) ranging between (1.04 ± 0.39) × 10^-11^ and (1.60 ± 0.37) × 10^-10^ M, which allowed us to propose the *E. histolytica* TATA-box sequence as 5′-(1: T) (2: A) (3: T/G) (4: T/G/A) (5: T/G/A) (6: A/G) (7: A) (8: A)-3′, where the numbers indicate the nucleotide position in TATA-box [19]. EhTRF1 has the ability to bind to the TATTTAAA-box as well, with a *K*_*D*_ value of (1.12 ± 0.16) × 10^-11^ M.

Determining the role of the closely related basal transcription factors TBP and TRF1 in the *E. histolytica* unicellular parasite will improve our understanding of the biology of this human parasite. As a first approach to unveiling the roles of these transcription factors, we determined the DNA-binding capacity of EhTBP and EhTRF1 for TATA variants, as well as for the GAAC core promoter element, through in vitro Electrophoretic Mobility Shift Assay (EMSA) experiments. Our results showed that both transcription factors have the capacity to bind to most of the TATA variants tested, but possess subtle differences in their *K*_*D*_ values. Remarkably, they have the ability to bind to the GAAC-boxes as well. Furthermore, the differential expression profiles of the *Ehtbp* and *Ehtrf1* genes were determined under different stress conditions, including serum depletion, heat-shock stress, and UV-irradiation. Additionally, we also determined their mRNA levels during the interaction of trophozoites with different mammalian cells, such as erythrocytes and Madin-Darby canine kidney (MDCK) target cells, as well as during the formation of amebic liver abscesses. Differential mRNA levels for both basal transcription factors were observed, suggesting that they have distinctive roles in these processes. Gene knockdown experiments in *E. histolytica* G3 trophozoites using the plasmid psAP-2 [32], unveiled the important role of *Ehtbp* in erythrophagocytosis.

## Methods

### *Entamoeba histolytica* Cell culture

Trophozoites of *E. histolytica* HM1:IMSS Clone A [33] were axenically cultured and maintained in either 15 ml glass tubes (Kimax, Mexico) or 25 cm^2^ flasks (Corning, Corning, NY, USA) with 13 ml or 20 ml of TYI-S-33 Diamond’s medium, respectively, at 37 °C. For gene silencing experiments, *E. histolytica* G3 trophozoites were cultured with or without G418 (Sigma-Aldrich, Saint Louis, MO, USA) added to the TYI-S-33 medium [32].

### Expression and purification of recombinant EhTRF1 and EhTBP proteins

Plasmids p*Ehtbp* [19] and p*cEhtrf1* [30] were transformed in *Escherichia coli* BL21(DE3)pLysS competent cells (Invitrogen, Carlsbad, CA, USA). Expression, purification and Western blot analysis of recombinant EhTBP (rEhTBP) and EhTRF1 (rEhTRF1) polypeptides were performed as described previously [30].

### Electrophoretic mobility shift assays and competition experiments

Electrophoretic mobility shift assays (EMSAs) were performed using different amounts (10 to 500 nM) of each purified rEhTBP or rEhTRF1 polypeptides in reaction mixtures containing 1 μg/μl of poly(dG-dC)·poly(dG-dC) in binding buffer (60 mM KCl, 1 mM each dithiothreitol, EDTA, spermidine and MgCl_2_, 10% glycerol, 12 mM Hepes, pH 7.9). The double-stranded oligonucleotide to be used as a probe (TATTTAAA oligonucleotide, TATA variants or GAAC-boxes) was added to the reaction mixture (20,000 cpm, 557 pM for each TATA-box, 64 pM for GAAC-box or 82 pM for GAAC-like box), which was previously labeled at its 5′-end with [γ-^32^P] ATP (3000 Ci/mmol, 10 μCi/μl, Perkin-Elmer, Waltham, MA, USA) with T4 polynucleotide kinase (Promega, WI, USA) and quantified with a Beckman LS-6500 liquid scintillation counter (Brea, CA, USA). EMSA assays were performed for 10 min at 4 °C. For competition experiments, 200-fold molar excess of unlabeled TATTTAAA probe or the different TATA-box variants or GAAC-boxes (Table 1) were used as specific competitors, while an unspecific competitor [poly(dG-dC)·poly(dG-dC), poly(dI-dC) or (dA-dT)_18 mer_] was used. In any case, selected competitors were added to reaction mixtures 10 min before the addition of labeled probes. When incubation time finished, all the reactions were electrophoresed on 6% nondenaturing polyacrylamide (PAGE) gels in 0.5× TBE (45 mM Tris-borate, 1 mM EDTA, pH 8.3). Gels were then vacuum-dried, and DNA-protein complexes were detected in a PhosphorImager system (Bio-Rad, Hercules, CA, USA). All experiments were done at least three times in duplicate with reproducible results.

**Table 1:** Sequences of the oligonucleotides used in EMSA experiments

Oligonucleotide	Sequence (5′-3′)
TATTTAAA (1)	AATTCTCTATTTAAAGAGAATT
TAgTgAAA (2)	AATTCTCTAgTgAAAGAGAATT
TATTggAA (3)	AATTCTCTATTggAAGAGAATT
TATTaAAA (4)	AATTCTCTATTaAAAGAGAATT
TATgTAAA (5)	AATTCTCTATgTAAAGAGAATT
gAgTTAAA (6)	AATTCTCgAgTTAAAGAGAATT
TAcTcAAA (7)	AATTCTCTAcTcAAAGAGAATT
cAcTcAAA (8)	AATTCTCcAcTcAAAGAGAATT
cAcTTAAA (9)	AATTCTCcAcTTAAAGAGAATT
TATTTttt (10)	AATTCTCTATTTtttGAGAATT
GAAC-box (Wild type)	AAGACAATGAACTAGAATG
GAAC-box^a^ (Mutated)	AAGACCTACGATAAGAATG
GAAC-like box^b^	AGGCGAACTACAAAAGAT

^a^Wild type and mutated GAAC-boxes are identical to those reported for *hgl*5 gene [33]

^b^This box was previously reported [17]

### Quantification of DNA-protein complexes

The quantification of DNA-protein complexes formed by rEhTBP and rEhTRF1 with the DNA-probes was performed by densitometry analysis using the Quantity One software package version 4.6.2 (Bio-Rad). Normalization was performed with free probe to correct the total radioactivity loaded in each gel lane [19, 30].

### Determination of dissociation constants of DNA-protein complexes

The *K*_*D*_ values for rEhTBP and rEhTRF1 polypeptides for the different TATA-boxes, wild type and mutated GAAC-boxes were determined by EMSA as previously described [19, 30, 34]. The dissociation constant value calculation is briefly described (Additional file 1).

### Determination of the *E. histolytica* growth curve

Two hundred thousand trophozoites from the mid-logarithmic growth phase were inoculated in each of two-glass tubes containing 13 ml of TYI-S-33 medium and incubated at 37 °C for 12, 18, 36, 72, 96 and 120 h. Then, cells were collected by centrifugation at 1800 *rpm* (652× *g*) in a 5810 R centrifuge (Eppendorf, Hamburg, Germany) at 4 °C. Cultured samples (50 μl) were used to determine the viable cell number using 0.4% Trypan blue dye (Bio-Rad) in 1× PBS and a Neubauer chamber. The remaining cells were kept in an ice bath and immediately used for total RNA isolation as described below.

### Culture of trophozoites in serum depletion condition

One million trophozoites collected at the exponential growth phase were cultured in 13 ml of TYI-S-33 medium with or without 20% bovine serum at 37 °C for 12 h as described previously [35]. These trophozoites were then checked for viability and processed for total RNA isolation. To observe the cellular morphology, cells contained in 0.5 ml of the culture were fixed with 2.5% (*v*/v) glutaraldehyde in 1× PBS for 15 min at RT, washed three times with 1× PBS by centrifugation, and observed through a light microscope (Olympus BH-2, Tokyo, Japan). Photomicrographs were obtained using a Pixera Penguin 600CL digital camera (Pixera, Santa Clara, CA, USA) coupled to the light microscope.

### Confocal microscopy

During the growth curve experiment, trophozoites were collected at each time point and washed with 1× PBS. Then, cells were grown on coverslips to be fixed with pre-warmed 4% paraformaldehyde (PFA) for 1 h at 37 °C and permeabilized with 0.5% Triton X-100 for 10 min at RT. Cells were carefully washed with 1× PBS and blocked with 10% bovine serum for 30 min at RT. Cells were incubated overnight at 4 °C with the anti-EhTBP primary antibody (1:400), followed by incubation with goat anti-rabbit TRITC labeled (1:100) secondary antibody (Zymed, Waltham, MA) for 1 h at 37 °C. The nucleus was stained with DAPI (Zymed) for 5 min at RT and the coverslips were carefully mounted with Vectashield antifading reagent (Vector Labs, Burlingame, CA, USA). The confocal image acquisition was performed using an LSM 700 microscope (Carl Zeiss, Oberkochen, Geremany). The captured images were analyzed and processed with the Zen 2009 lite edition software (Carl Zeiss). The same procedure was used for cells obtained in the presence or absence of serum for confocal microscopy analysis.

### Heat-shock of trophozoites at 42 °C

One million trophozoites collected at the mid-log phase were cultured at 37 °C or 42 °C in 15 ml glass tubes containing 13 ml of complete TYI-S-33 medium for 4 h [36]. A sample of 0.5 ml was separated before the RNA isolation for the examination of the cellular morphology as described above, and total RNA extraction was carried out immediately and stored at -80 °C until use. As a control indicator of heat-shock stress, the gene expression level of *Ehhsp70* was measured by RT-qPCR [36].

### Exposure of trophozoites to UV irradiation

Two million trophozoites harvested at exponential growth phase were transferred to Petri dishes (100 mm in diameter). Following their adherence to the surface of the petri dish, the culture medium was removed and the cells were immediately exposed to 254 nm UV light (150 J/m^2^) for eight seconds as described [37] using a Stratalinker 1800 (Stratagene, San Diego, CA, USA). After exposure, 20 ml TYI-S-33 medium prewarmed at 37 °C was added to the plates. The cells were detached by placing them on the ice after 5, 15 and 30 min. The cells were processed later for the viable cell count and total RNA isolation. Cells unexposed to UV irradiation were used as a control. The *Ehblm*, *Ehrad54* and *Ehpcna* DNA repair genes were included in this experiment as reference genes [38, 39].

### Erythrophagocytosis

Fresh human erythrocytes (O type Rh negative) were washed three times with sterile filtered 1× Alsever isotonic solution (0.42% NaCl, 0.8% sodium citrate, 0.055% citric acid and 2% D-glucose) and incubated with trophozoites (50:1 erythrocytes/trophozoites ratio) in serum free TYI-S-33 medium at 37 °C for 2, 5, 10, 15 and 30 min [33]. After incubation, cold distilled water was added to burst the non-ingested erythrocytes and trophozoites were collected for total RNA isolation and counting of ingested erythrocytes by staining cells with 8.4 mM diaminobenzidine. For counting, a population of 100 trophozoites was selected randomly for all time points. Images were obtained as mentioned before.

### MDCK cell monolayer destruction by trophozoites

Initially, MDCK epithelial cells were cultured in DMEM medium supplemented with penicillin (100 I.U./ml; in vitro), streptomycin (100 mg/ml, in vitro), 10% fetal bovine serum (Gibco, Carlsbad, CA, USA) and insulin (0.08 U/ml, Eli Lilly, Indianapolis, IN, USA) at 37 °C and 5% CO_2_. One and a half million trophozoites were carefully deposited on a confluent fresh MDCK epithelial cell monolayer and incubated at 37 °C in serum free TYI-S-33 medium for 5, 15 and 30 min. After incubation, trophozoites were detached by repetitive washing with ice-cold 1× PBS and collected for total RNA extraction. For the evaluation of MDCK cell destruction by trophozoites, cells were fixed with 2.5% glutaraldehyde in 1× PBS for 15 min at RT and washed with 1× PBS as previously described [40]. Cells were then stained with 1% methylene blue in 0.01% sodium borate buffer (0.2 M boric acid and 0.05 M sodium tetraborate, pH 8.7) for 10 min at RT and compared with intact MDCK cell monolayer. Finally, stained monolayers were washed three times with 1× PBS and left to air dry the plate to capture images using the Epson V500 Photo scanner. The percentage of monolayer destruction was measured using ImageJ software.

### Generation of amoebic hepatic liver abscesses in hamsters

To induce the formation of liver abscesses, Syrian gold hamsters (*Mesocricetus auratus*) were anesthetized using 45 mg/kg of sodium pentobarbital, and intraportally injected with four million trophozoites collected from the mid-log phase. After seven days of post-inoculation, animals were sacrificed by giving an overdose of sodium pentobarbital following the guidelines of the 2000 AVMA Panel of Euthanasia. Livers were dissected under sterile conditions for examination of abscesses. The abscesses were cut into small pieces to facilitate the release of virulent trophozoites into 13 ml pre-warmed TYI-S-33 medium as described [41]. Once the cultures were confluent, cells were harvested for total RNA isolation. Three hamsters were used for each experiment and three independent experiments were performed. Animal management was supervised by a licensed veterinarian in accordance with the principles set forth in the NIH guide for the care and use of laboratory animals. These experiments were approved by the Institutional Animal Care and Use Committee (IACUC/Bioethics) of Cinvestav-IPN.

### Gene silencing using *E. histolytica* G3 trophozoites

A fragment of 400 bp from the translation start codon (ATG) of *Ehtbp* gene was amplified using specific primers (Additional file 2: Table S1) and cloned into a pJET1.2 vector (Thermo Scientific, Waltham, MA, USA). DNA recognition sites for *Stu*I and *Sac*I at 5′- and 3′-ends, respectively, were included for cloning this fragment into the psAP2 vector [32]. G3 trophozoites were cultured in 6-well plates (Corning) and 20 μg of the plasmid psAP2-*Ehtbp* were transfected using Superfect liposomes (Qiagen, North Rhine-Westphalia, Hilden, Germany). After 48 h of transfection, 1 μg/ml of G418 (Sigma-Aldrich) was added and gradually increased up to 10 μg/ml to obtain stable transfectants. As a control, the empty vector was transfected and stable transfectants were obtained. To measure the rate of erythrophagocytosis and to obtain growth curve, we followed the procedure as described above.

### Quantitative real-time PCR (RT-qPCR)

Total RNA isolation was performed with Trizol reagent (Invitrogen) following the manufacturer’s instructions and quantified with a Nanodrop system 2000c (Thermo Scientific). Total RNA (5 μg) was DNase I-treated (Thermo Scientific) and samples of 100 ng were subjected to cDNA synthesis at 50 °C for 30 min and specific genes were amplified by PCR using the SYBR FAST One-Step RT-qPCR (Kapa Biosystems, Wilmington, MA, USA) under the following conditions: initial denaturation at 95 °C for 5 min, followed by 40 cycles of denaturation at 95 °C for 10 s, annealing at 58 °C for 30 s and extension at 72 °C for 20 s using an Eco™ Real-Time PCR System (Illumina, San Diego, CA, USA). The expression level of target genes was normalized with the house keeping *Eh40Ss2* gene (GenBank: EHI_020280), and then the relative gene expression was measured using the equation 2^−∆∆*Cq*^ [42] with the Eco Real-Time PCR System software version 4.02. Appropriate no added reverse transcriptase and non-template controls were included in each of 48-well reaction plates. The oligonucleotides used in RT-qPCR assays are listed (Additional file 2: Table S1). For all statistical analysis, the Graphpad Prism version 6.0 program was used. The analysis was performed using two tailed unpaired Student’s t-test and significant difference values were shown as mean ± standard deviation of three biological replicates, each done in triplicate.

## Results

### EhTBP and EhTRF1 have the capacity to bind to different TATA-variants in vitro

**Fig. 1:**
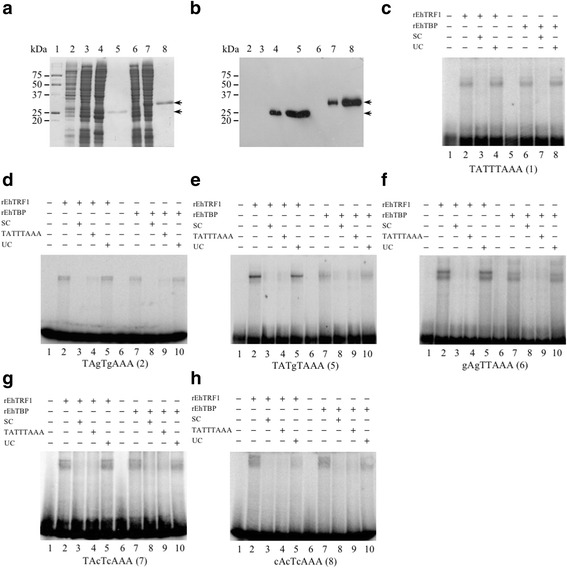
EhTBP and EhTRF1 have DNA-binding activity to different TATA variants in vitro. Recombinant EhTBP and EhTRF1 were expressed in *E. coli* BL21(DE3) pLysS, purified by IMAC and analyzed by electrophoresis as described in Methods. **a** SDS-PAGE (12%) gel analysis of extracts from bacteria transformed with pRSET A (Lane 2), pc*Ehtrf1* (Lanes 3 and 4), p*Ehtbp* (Lanes 6 and 7). Protein expression was induced with 1 mM IPTG for rEhTRF1 and rEhTBP (Lanes 4, and 7, respectively). rEhTRF1 and rEhTBP purified proteins (arrows) are shown in Lanes 5 and 8, respectively. **b** Western blot of the gel shown in **a** using anti-His tag monoclonal antibodies. EMSA assay to determine the DNA-binding activity of purified rEhTRF1 and rEhTBP using different probes: (**c**) TATTTAAA(1); (**d**) TAgTgAAA(2); (**e**) TATgTAAA(5); (**f**) gAgTTAAA(6); (**g**) TAcTcAAA(7); (**h**) cAcTcAAA(8). *Abbreviations*: UC, unspecific competitor; SC, specific competitor

We previously described the DNA-binding capacity of rEhTBP for different TATA variants, as well as the binding capacity of rEhTRF1 for the TATTTAAA-box [19, 30]. To continue with the characterization of these transcription factors (rEhTBP and rEhTRF1), they were expressed in *E. coli* (Fig. 1a, lanes 4 and 7, respectively) and purified by Immobilized Metal Affinity Chromatography (IMAC) (Fig. 1a, lanes 5 and 8, respectively). Western blot analysis confirmed that they are his-tagged proteins (Fig. 1b). To determine whether they were biologically active, they were analyzed in EMSA assays using the TATTTAAA-box as a probe [43]. Recombinant EhTRF1 and EhTBP proteins showed TATTTAAA-box binding capacity (Fig. 1c, lanes 2 and 6, respectively). Complexes formed by these two proteins were specific as their formation was completely abolished by 200-fold molar excess of unlabeled probe (Fig. 1c, lanes 3 and 7), but remained intact when an excess of unspecific competitor was used (Fig. 1c, lanes 4 and 8). Then, EMSA assays were performed using different TATA variants that have not been previously studied (Fig. 1d-h). Assays performed with the TAgTgAAA(2) variant showed a slightly higher amount of DNA-protein complexes formed by rEhTRF1 as compared to rEhTBP (Fig. 1d, lanes 2 and 7, respectively). A similar behavior was observed for TATgTAAA(5) (Fig. 1e, lanes 2 and 7, respectively), and gAgTTAAA(6) (Fig. 1f, lanes 2 and 7). The formation of DNA-protein complexes was abolished when a 200-fold molar excess of unlabeled specific TATA variant was used in competition experiments for both rEhTRF1 and rEhTBP polypeptides (Fig. 1d-h, lanes 3 and 8, respectively). The specific competition was obtained when the TATTTAAA-box was used as a competitor for the different TATA variants tested (Fig. 1d-h, lanes 4 and 9). When a 200-fold molar excess of unspecific competitor was used in competition experiments, the formation of DNA-protein complexes was unaffected (Fig. 1d-h, lanes 5 and 10). The formation of DNA-protein complex for cAcTTAAA (9) was seen only with rEhTRF1 (Additional file 3: Figure S1).

### EhTBP and EhTRF1 are able to bind the GAAC-boxes in vitro

**Fig. 2:**
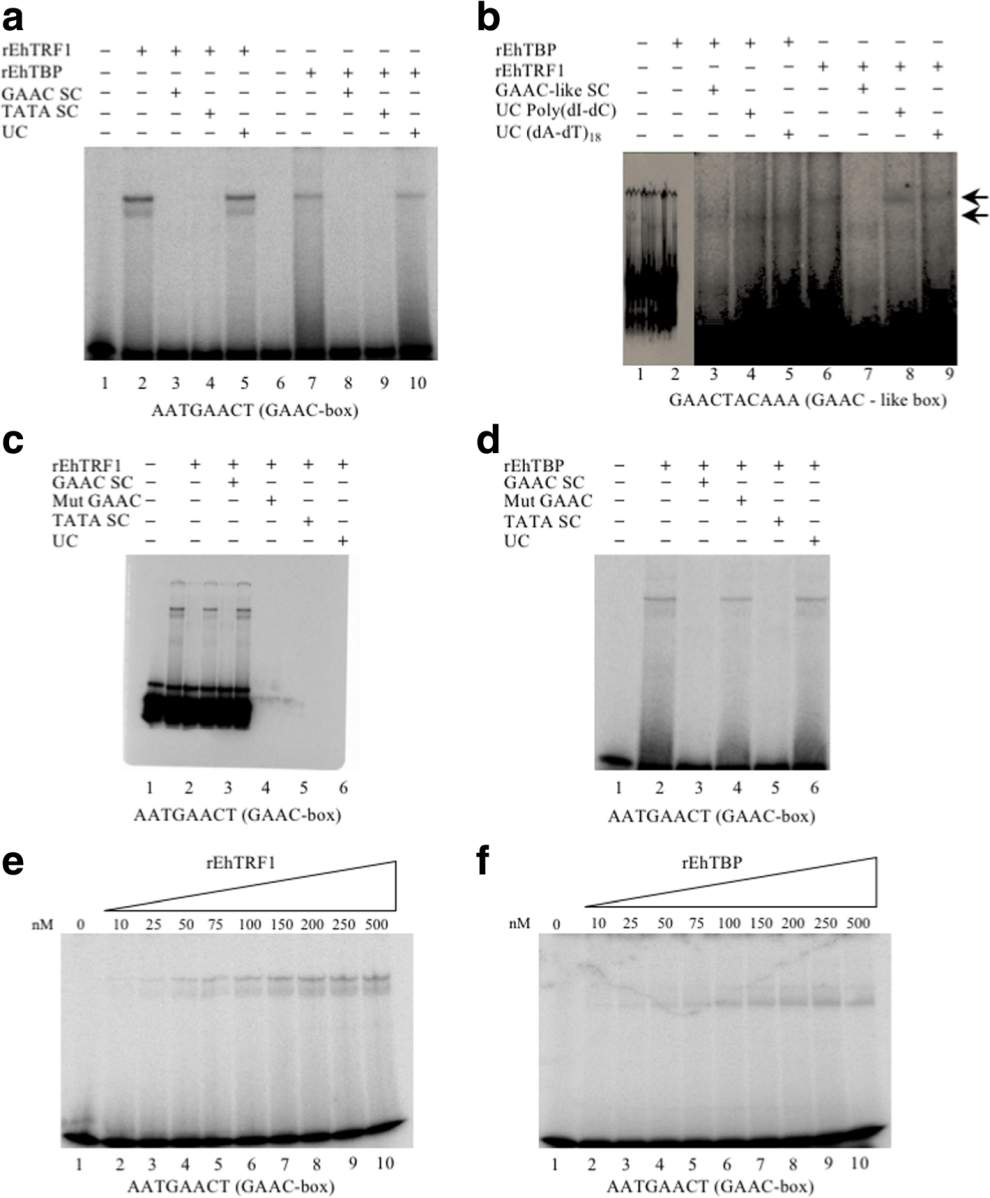
EhTRF1 and EhTBP are GAAC-box binding proteins (GBPs). **a** EMSA using the GAAC-box. Formation of rEhTRF1-GAAC and rEhTBP-GAAC complexes was competed by the unlabeled GAAC-box and TATTTAAA-box (Lanes 3 and 4, and 8 and 9, respectively). **b** EMSA using the GAAC-like box. The rEhTBP or rEhTRF1 with GAAC-like box forming complex (Lanes 2 and 6). Specific competition with 200-fold molar excess of unlabeled probe (Lanes 3 and 7) or unspecific competition with poly(dI-dC) or (dA-dT)_18 mer_ oligonucleotide (Lanes 4 and 8, and Lanes 5 and 9, respectively). **c**, **d** Mutated GAAC-box was unable to compete the formation of rEhTRF1-GAAC-box and rEhTBP-GAAC-box complexes (Lane 4). **e**, **f** Quantification of the GAAC-box protein complexes as a function of increasing concentrations of rEhTRF1 (**e**) or rEhTBP (**f**) by EMSA experiments

**Fig. 3:**
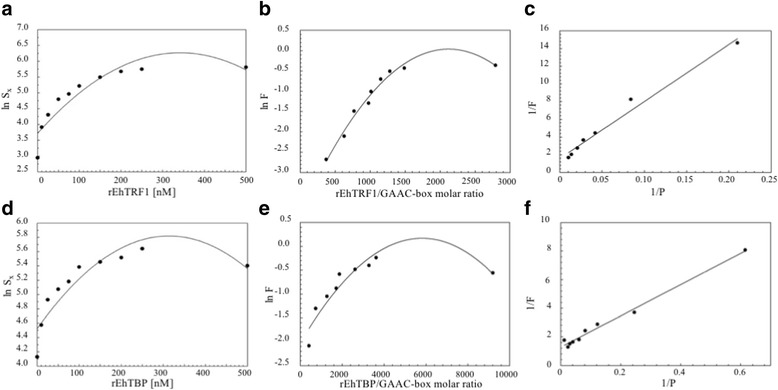
Graphical representation of the data obtained in the quantification of DNA-protein complexes shown in Fig. 2 (**e** and **f). a, d** Data corresponds to the natural logarithm of the radioactivity present in the DNA-protein complexes (*ln S*_*x*_) as a function of the protein concentration *x*. Dots correspond to experimental data. The continuous line is the graph predicted by the second-degree polynomial function. **b**, **e** Mathematical relationships shown by the natural logarithm of the fraction (*F*) of DNA probe in GAAC-box-protein complexes and the rEhTRF1/GAAC-box molar ratios (**b**) and rEhTBP/GAAC-box molar ratio (**e**). Dots correspond to experimental data. Continuous line is the graph predicted by the second-degree polynomial function. **c**, **f** Mathematical relationships shown by the reciprocal of *F* (*1/F*) and the reciprocal of the total active uncomplexed protein *P* (*1/P)* for rEhTRF1 and rEhTBP, respectively. Dots correspond to experimental data. The continuous line is the graph predicted by the linear eq. *1/F* = *K*_*D*_ *(1/P) + 1*, where its slope corresponds to the *K*_*D*_ value. The arrow indicates the complex with GAAC-like box

To test the possibility that EhTRF1 and EhTBP are two putative GAAC-box binding proteins (GBPs), the DNA-binding capacities of these two proteins were determined by EMSA experiments (Fig. 2a, lanes 2 and 7, respectively). The complexes formed by these transcription factors with the GAAC-box were specific as they disappeared with 200-fold molar excess of unlabeled GAAC-box (Fig. 2a, lanes 3 and 8). The formation of these DNA-protein complexes was also competed with a 200-fold molar excess of the TATTTAAA-box (Fig. 2a, lanes 4 and 9). In addition, the binding capacity of rEhTBP and rEhTRF1 for the GAAC-like box was also tested. The former bound the GAAC-like box, but the complex was barely competed by the specific competitor (Fig. 2b, lanes 2 and 3, respectively) and was not abolished with either 200-fold molar excess of poly(dI-dC) or (dA-dT)_18 mer_ (Fig. 2b, lanes 4 and 5, respectively). The latter formed a complex with the GAAC-like box, which was specifically competed (Fig. 2b, lanes 6–9). Moreover, the formation of the GAAC-rEhTBP or GAAC-rEhTRF1 complexes was not abolished when a 200-fold molar excess of the mutated GAAC-box was used (Fig. 2c, d, lane 4). The *K*_*D*_ values of rEhTBP and rEhTRF1 for the GAAC-box (Fig. 3a-f) were (1.90 ± 0.16) × 10^-11^ M and (7.94 ± 1.44) × 10^-11^ M, respectively (Table 2).

**Table 2:** Dissociation constants (*K*_*D*_) of rEhTBP and rEhTRF1 for different TATA variants and GAAC-box

DNA sequence	rEhTBP	rEhTRF1
TATTTAAA (1)	(2.07 ± 0.49) × 10^-11^ M	(5.29 ± 0.98) × 10^-11^ M
TAgTgAAA (2)	(3.98 ± 0.16) × 10^-11^ M	(1.30 ± 0.51) × 10^-11^ M
TATTggAA (3)	(3.57 ± 0.13) × 10^-11^ M	(1.35 ± 0.88) × 10^-11^ M
TATTaAAA (4)	(4.85 ± 3.06) × 10^-12^ M	(3.98 ± 1.96) × 10^-12^ M
TATgTAAA (5)	(1.69 ± 1.37) × 10^-12^ M	(6.74 ± 2.25) × 10^-12^ M
gAgTTAAA (6)	(4.73 ± 2.91) × 10^-12^ M	(6.85 ± 4.77) × 10^-12^ M
TAcTcAAA (7)	(2.76 ± 1.48) × 10^-12^ M	(2.98 ± 1.37) × 10^-11^ M
cAcTcAAA (8)	(3.80 ± 1.46) × 10^-11^ M	(3.08 ± 1.15) × 10^-11^ M
cAcTTAAA (9)	NB	(9.72 ± 4.88) × 10^-12^ M
TATTTttt (10)	(3.40 ± 1.64) × 10^-12^ M	(2.54 ± 1.37) × 10^-11^ M
GAAC-box	(1.90 ± 0.16) × 10^-11^ M	(7.94 ± 1.44) × 10^-11^ M

*Abbreviation*: *NB* No binding activity for this DNA sequence

### *Ehtbp* gene is overexpressed in nutrient and serum depletion

**Fig. 4:**
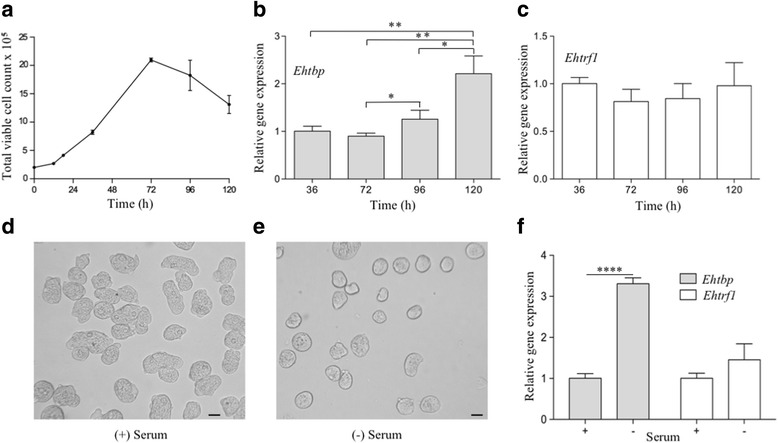
*Ehtbp* is upregulated during nutrient deprivation. **a** Growth curve of trophozoites at 12, 18, 36, 72, 96 and 120 h. **b**, **c** Relative gene expression levels of *Ehtbp* (**b**) and *Ehtrf1* (**c**) at different times of growth curve. Statistical significance (Student’s t-test) defined as *P < 0.05, **P < 0.001. **d**, **e** Images of trophozoites grown in TYI-S-33 medium for 12 h at 37 °C with serum (**d**) or without serum (**e**). Photomicrographs were obtained with a magnification of 200×. **f** RT-qPCR analysis of *Ehtbp* and *Ehtrf1* genes. Statistical significance was determined using the Student’s t-test (****P < 0.0001). *Scale-bar*: 10 μm

The expression of *Ehtbp* and *Ehtrf1* was determined at several points of the growth cycle of *E. histolytica* in TYI-S-33 medium (Fig. 4). The exponential growth phase began at 36 h and finished at 72 h. After this time, we observed a decline of 13.0% and 37.4% in the viable cell count at 96 and 120 h, respectively, in comparison to the maximum value reached at 72 h (Fig. 4a). Since there were no changes in the levels of *Ehtbp* and *Ehtrf1* mRNAs at 12, 18 and 36 h (data not shown), the 36 h sample was selected as the reference point in our RT-qPCR analysis. The *Ehtbp* gene expression level did not change at 72 h as well. However, it increased 26% and 121% at 96 h and 120 h, respectively (Fig. 4b). These two points correspond to the decline or death phase of growth curve (Fig. 4a). In the case of *Ehtrf1*, its mRNA expression level did not significantly change in the growth curve (Fig. 4c). Hence, *Ehtbp* could play a role in the response to nutrient depletion in trophozoites.

To explore the putative role of *Ehtbp*, cells were grown in serum-depleted media (Fig. 4). Under this stress condition, cells changed their amoeboid morphology (Fig. 4d) to a round shape (Fig. 4e). In both cases, cell viability was 95%. When subjected to serum depletion, *Ehtbp* mRNA levels showed a sharp increase, up to 231% in comparison to normal conditions. Meanwhile, *Ehtrf1* expression did not show any significant changes (Fig. 4f).

**Fig. 5:**
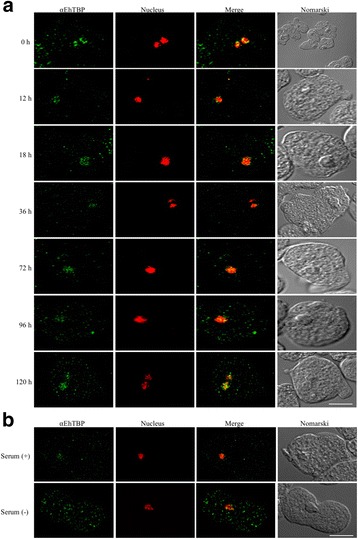
Immunolocalization of EhTBP during the growth curve and serum depletion. **a** Cells were grown at the indicated times and immunostained with rabbit polyclonal anti-rEhTBP antibodies (green channel). DNA was stained with DAPI (red channel). **b** Trophozoites were grown in the presence or absence of serum and processed as in (**a**). *Scale-bar*: 10 μm

We also immunolocalized EhTBP at the same time points of the growth curve and during serum depletion (Fig. 5 and Additional file 4: Figure S2). In the initial growth phase (0 to 36 h), EhTBP was mainly localized in the nucleus, while at 72 h, it was also observed in the cytoplasm. During decline phase at 96 and 120 h, this protein was detected both in the cytoplasm and nucleus (Fig. 5a). During serum depletion, EhTBP was localized both in cytoplasm and nucleus, while in the presence of serum, it was only observed in the nucleus (Fig. 5b).

### *Ehtrf1* gene expression is negatively regulated during UV-irradiation and heat-shock stress

**Fig. 6:**
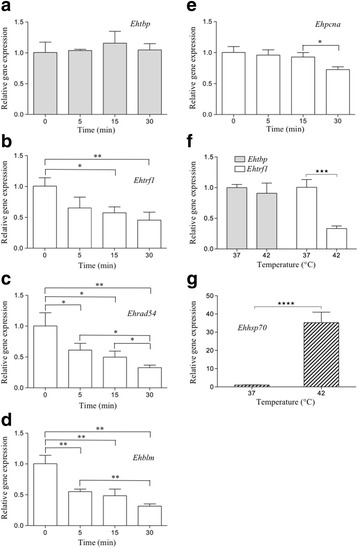
*Ehtrf1* is downregulated in response to UV irradiation and heat-shock stress. UV-irradiation **a**-**e** Relative gene expression of *Ehtbp* (**a**), *Ehtrf1* (**b**), *Ehrad54* (**c**), *Ehblm* (**d**) and *Ehpcna* (**e**) normalized with the endogenous gene control *Eh40Ss2*. **f** Gene expression analysis of *Ehtbp* and *Ehtrf1* by RT-qPCR in trophozoites cultured at 37 °C and 42 °C as described. **g** As a control of heat-shock response, the expression level of *Ehhsp70* gene was determined. No change in morphology was observed in both cases. Significant values are marked with asterisks (Student’s t-test, *P < 0.05, **P < 0.001, ***P < 0.0005)

Transcription of *Ehtbp* remained unchanged up to 30 min following UV irradiation (Fig. 6a). However, *Ehtrf1* transcription significantly diminished to 0.57 and 0.45 at 15 and 30 min after UV exposure, respectively, in comparison to non-irradiated cells (Fig. 6b). We determined the transcription levels of *Ehrad54*, *Ehblm* and *Ehpcna* genes in this experiment as reference genes because they are UV-responsive genes and are involved in DNA repair [38, 39]. In the case of *Ehrad54*, it showed a decrease to 0.61, 0.49 and 0.32 transcription level at 5, 15 and 30 min, respectively (Fig. 6c). *Ehblm* also showed a decrease to 0.55 and 0.31 at 5 and 30 min after UV irradiation, respectively (Fig. 6d). Finally, the *Ehpcna* mRNA level diminished to 0.72 after 30 min of UV exposure (Fig. 6e). Results obtained for *Ehrad54* mRNA level were similar to those previously reported [38]. In contrast to this, while the *Ehblm* mRNA level decreased in our case, the EhBLM protein level increased 2-fold and 34-fold at 5 and 30 min in cytoplasmic extracts, respectively [39]. It is important to note that cell viability after UV-irradiation was 97% by trypan blue exclusion assay.

Trophozoites that were incubated at 42 °C for 4 h showed no change in their cellular morphology (data not shown). The cell viability was 95% following heat-shock. Trophozoites in this stress condition showed no change in the *Ehtbp* mRNA transcription level. However, the *Ehtrf1* mRNA level was reduced to 33% in comparison to its transcription under standard growth conditions (Fig. 6f). To corroborate the heat-shock stress response, the gene expression level of *Ehhsp70* was determined, displaying 35-fold increase at 42 °C (Fig. 6g).

### *Ehtrf1* and *Ehtbp* gene expression levels are downregulated during erythrophagocytosis

**Fig. 7:**
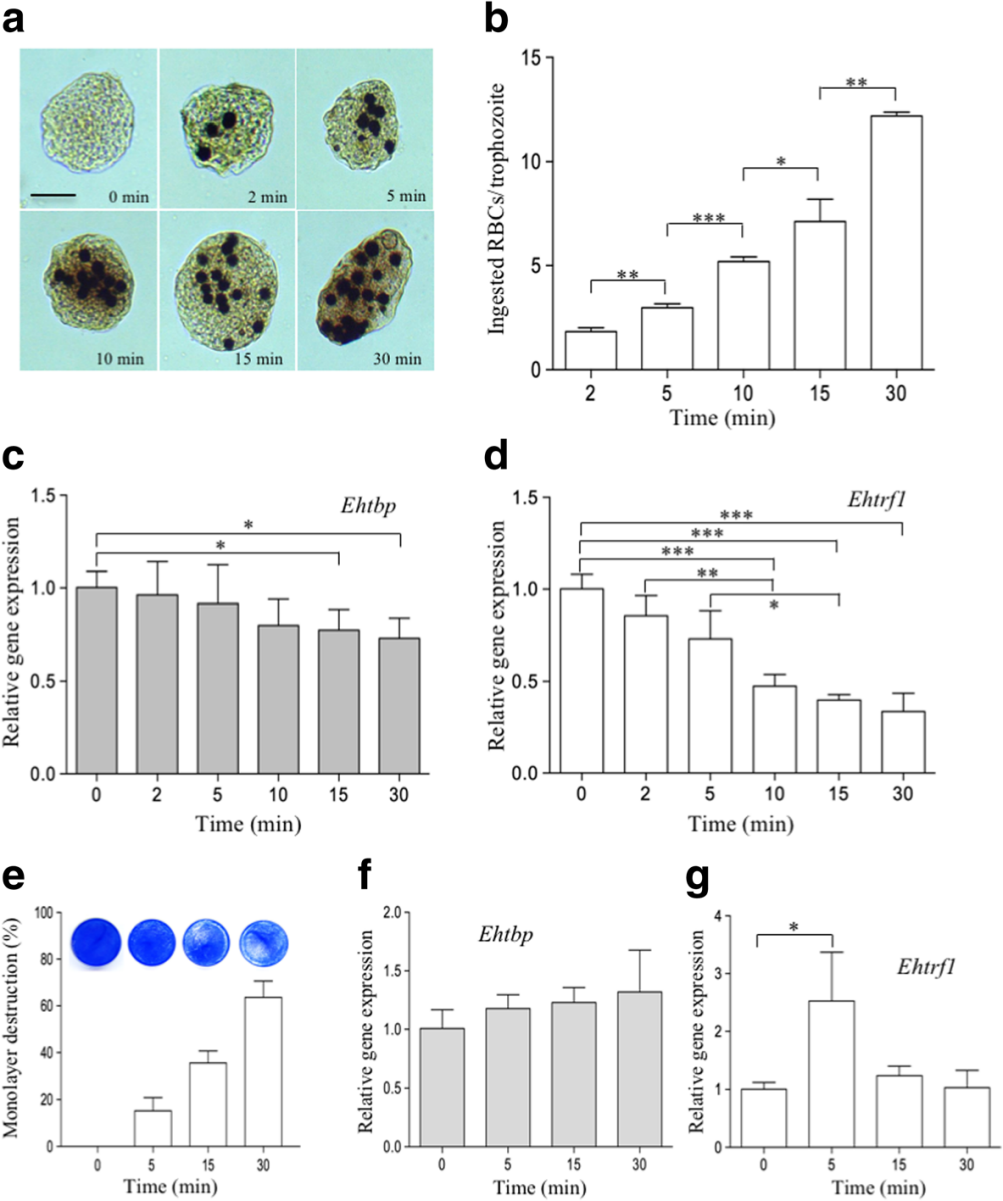
Gene expression analyses of *Ehtrf*1 and *Ehtbp* genes of *E. histolytica* trophozoites during erythrophagocytosis and MDCK monolayer destruction and amoebic abscess formation. **a**-**d** Erythrophagocytosis assay. **a** Representative photomicrographs showing the accumulation of ingested erythrocytes in trophozoites at different time points. Photomicrographs were taken at 200× magnification. **b** Graph showing the average number of ingested erythrocytes by trophozoites. **c**, **d** Gene expression levels of *Ehtbp* (**c**) and *Ehtrf1* (**d**). **e**-**g** MDCK cell monolayer destruction by trophozoites. **e** Images of representative culture plates of stained MDCK cell monolayers destroyed by live trophozoites interacting for 5, 15 and 30 min. An intact MDCK cell monolayer is shown (0 min) for comparison. **f**, **g** RT-qPCR analysis of *Ehtbp* and *Ehtrf1* genes, respectively. Statistical analysis with significant difference are marked with asterisks (Student’s t-test, *P < 0.05, **P < 0.001, ***P < 0.0003). *Scale-bar*: 10 μm

During erythrophagocytosis, trophozoites ingested increasing numbers of RBCs as a function of time, reaching an average of 12 ingested erythrocytes per trophozoite in 30 min (Fig. 7a, b). To determine the count, one hundred randomly selected trophozoites were counted at each time interval and the data were plotted to calculate the average of ingested erythrocytes per trophozoite (Fig. 7b). Interestingly, the *Ehtbp* mRNA transcription level slightly diminished to 0.77 and 0.73 at 15 and 30 min, respectively, while *Ehtrf1* transcription level significantly decreased to 0.47, 0.39 and 0.33 at 10, 15 and 30 min, respectively (Fig. 7c, d, respectively).

### *Ehtrf1* gene expression is transiently upregulated during MDCK cell monolayer destruction

The other virulence condition tested was the incubation of trophozoites with MDCK epithelial cell monolayers at different times. An intact MDCK cell monolayer was homogeneously stained with methylene blue (Fig. 7e, 0 min). However, when trophozoites were incubated with the cell monolayer, the destruction was evidenced by clear unstained areas, which increased with time (Fig. 7e). The percentage of monolayer destruction was 15.5%, 35% and 64% at 5, 15 and 30 min respectively (Fig. 7e). In these conditions, the *Ehtbp* transcription level remained unchanged through all the tested time intervals (Fig. 7f). However, the *Ehtrf1* transcription level showed a transient increase up to 2.5-fold at 5 min of interaction with MDCK cell monolayer, decreasing to its normal transcription level at 15 and 30 min in comparison to the 0 min condition (Fig. 7g).

### *Ehtrf1* is downregulated in trophozoites isolated from amoebic hepatic abscesses

**Fig. 8:**
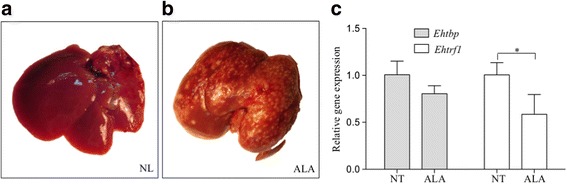
Gene expression analyses of *Ehtrf1* and *Ehtbp* genes of *E. histolytica* trophozoites during amoebic abscess formation. **a**, **b** Images of normal liver (NL) and with a number of amoebic liver abscesses (ALA), respectively. **c** Gene expression analysis of *Ehtbp* and *Ehtrf1* genes in trophozoites grown in normal conditions (NT) or isolated from amoebic liver abscesses (ALA). Statistical analysis with significant difference is marked with an asterisk (Student’s t-test, *P < 0.05)

We determined the mRNA levels of *Ehtbp* and *Ehtrf1* before and after the formation of liver abscesses in hamsters. After one week in the liver, trophozoites formed a huge number of small amoebic liver abscesses (ALA) (Fig. 8b) in comparison to normal liver (NL) (Fig. 8a). Trophozoites that were recovered by shredding the liver into small pieces and incubation for 3 to 4 days in TYI-S-33 medium showed no change in the transcription level of *Ehtbp* in comparison to trophozoites that had been kept in laboratory conditions (NT) for at least four months (Fig. 8c). However, the *Ehtrf1* mRNA level significantly diminished to 0.58 after recovery from liver abscess (Fig. 8c). Therefore, the combined transcriptional expression analysis (erythrophagocytosis, MDCK cell destruction and liver abscess) reveals that the *Ehtrf1* gene might have a role in virulence conditions.

### *Ehtbp* gene knockdown affects the rate of erythrophagocytosis in *E. histolytica* G3 trophozoites

**Fig. 9:**
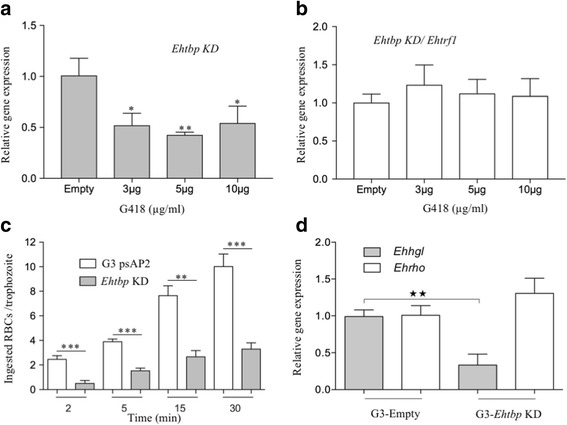
*Ehtbp* gene knockdown using G3 trophozoites. **a** Gene expression analysis of *Ehtbp* in knocked down (KD) cells grown at 3, 5, 10 μg/ml G418, using transfected cells with empty vector as a control. **b** Gene expression analysis of *Ehtrf1* gene in *Ehtbp* knockdown cells. **c** Number of ingested erythrocytes at 2, 5, 15 and 30 min in control G3 cells and 10 μg/ml G418 *Ehtbp* knockdown cells. **d** Gene expression analysis of two genes containing the GAAC motif (*Ehhgl*, *Ehrho*) in *Ehtbp* KD cells. Significant difference was measured using Student’s t-test and marked with asterisks (*P < 0.05, **P < 0.002, ***P < 0.0004)

In higher eukaryotes, TBP is considered vital for gene regulation [28]. To evaluate the importance of *Ehtbp*, we used G3 trophozoites and psAP2 vector for gene knockdown. Stable transfectants of *Ehtbp* were obtained by incubation with G418 at 3, 5 and 10 μg/ml for at least two months. Through RT-qPCR analysis, we observed a 50% decrease in *Ehtbp* transcription across all tested drug concentrations in comparison to control cells transfected with the empty plasmid (Fig. 9a). To characterize the phenotype of *Ehtbp* KD G3 trophozoites, we obtained the growth curves of G3 cells, G3 transfected with psAP2 empty vector, and G3 *Ehtbp* KD cells in TYI-S-33 medium (Additional file 5: Figure S3). We did not observe any growth defects or morphological changes in trophozoites under this condition. In serum depletion experiments with G3 *Ehtbp* KD trophozoites, we did not observe morphological changes in cells as well (data not shown). It has been previously reported that closely related genes might also be silenced when the primary target is silenced using this strategy [44], but we observed no change in the transcription of *Ehtrf1* in the *Ehtbp* knockdown strain (Fig. 9b)*.* The *Ehtrf1* gene knockdown was unsuccessful after several attempts using different strategies. Subsequently, we performed erythrophagocytosis assays to evaluate whether the silencing of *Ehtbp* had any impact on virulence (Fig. 9c). The assay was carried out with the *Ehtbp* knockdown trophozoites grown at 10 μg/ml G418 and cells transfected with empty vector as a control. The mean number of ingested erythrocytes was gradually increased from 2 (2 min) to 10 (30 min) in G3 control cells (Fig. 9c), while in the case of *Ehtbp* knockdown cells a lower mean number of ingested erythrocytes was observed, from 1 (at 2 min) to 4 (at 30 min) (Fig. 9c), indicating that the knocking down of *Ehtbp* gene reduced the rate of erythrophagocytosis. Transcription of the *Ehvps32* and *EhrabB* genes previously reported as being involved in phagocytosis was also evaluated in *Ehtbp* knockdown cells [45, 46]. However, both genes did not show any significant change in their mRNA levels (Additional file 5: Figure S3b).

Using the software described by Meneses et al. [16], we selected two genes containing only the GAAC motif to measure their mRNA levels by RT-qPCR in *Ehtbp* knockdown cells. The *Ehrho* (EHI_190440) gene promoter contains a GAAC-box at -63 to -56, while the *Ehhgl* (EHI_012270) promoter has the GAAC box at -57 to -50 from the corresponding theoretical transcription start sites. We observed no changes for the *Ehrho* gene. However, the mRNA amount of *Ehhgl* decreased 67% (Fig. 9d). These differences indicate the *Ehhgl* is being regulated by *Ehtbp*, while *Ehrho* gene might be controlled by other transcription factors.

## Discussion

In the present study, the DNA-binding activity of rEhTBP and rEhTRF1 polypeptides for different TATA variants was determined (Fig. 1 and Additional file 3: Figure S1). Remarkably, rEhTRF1 bound all the TATA variants assayed, while rEhTBP bound all but the cAcTTAAA (9) variant. *K*_*D*_ values were in the range of 10^-12^ M to 10^-11^ M for both transcription factors (Table 2, Additional file 6: Table S2, and Additional file 7: Table S3). Thus, in combining the results obtained before [19, 30] with those obtained in this study, the proposed *E. histolytica* TATA-box motif is defined as follows: 5′-(1: T/G/C) (2: A) (3: T/G/C) (4: **A**/T/G) (5: A/T/G/C) (6: A/T/G) (7: A/T) (8: A/T), where numbers in parenthesis indicate the position in TATA sequence, and the nucleotide in bold was only assayed for rEhTBP. Remarkably, the presence of two C’s in positions 1 and 3 abolished the DNA-binding capacity of rEhTBP only, which was rescued when a third C was in position 5.

In addition to the TATA-box and Inr sequences, a third unusual core promoter element named GAAC element has only been reported in *E. histolytica* [4, 47]. EMSA analysis revealed that both rEhTBP and rEhTRF1 bound the GAAC-box (Fig. 2) with *K*_*D*_ values of 10^-11^ M (Table 2). This element was identified in 31 gene promoters of 37 genes studied. Furthermore, other studies have confirmed that the GAAC-box controls both the expression rate and location of the transcription initiation start site [4]. A nuclear GAAC-box binding protein (GBP) of 29 kDa has been detected, but its identity remains unknown [47]. As the molecular masses of EhTBP and EhTRF1 are 26 kDa and 24 kDa, respectively, both proteins might be feasible candidates of GBPs, recruiting to promoters the TFIID complex and other basal transcription factors to assemble the pre-initiation complex [48]. Furthermore, both transcription factors showed binding capacity to the GAAC-like box, which has been found widely distributed in gene promoters of *E. histolytica* [17]. This is the first experimental study to demonstrate the binding capacity of the EhTBP and EhTRF1 transcription factors to both GAAC and GAAC-like boxes.

In higher eukaryotes, TBP is involved in transcription mediated by the three RNA polymerases. TBP-related factors have evolved in species as a form of specialization that allows the expression of sets of genes for determining biological functions. However, they have only been found in multicellular organisms possessing specialized cells, e.g. spermatids and oocytes. It seems feasible that the different sets of genes that *E. histolytica* employs to perform its specialized functions, including adhesion, host cell lysis, phagocytosis [33, 49], trogocytosis [50], encystment, response to stress conditions, etc. are under the control of master genes that coordinately regulates them. Several studies have allowed the identification of stress-related transcription factors in response to external perturbations. However, the role of basal transcription factors under those stringent conditions remains unclear. In this work, we determined the *Ehtbp* and *Ehtrf1* mRNA levels under different conditions intimately linked to parasite’s virulence, as well as those related to stress responses.

The first condition tested was throughout the growth in laboratory conditions. During the decline phase, abnormal spherically shaped cells were observed, losing surface adherence and amoeboid movement (data not shown). Interestingly, the *Ehtbp* mRNA level was nearly doubled at 120 h in comparison to the previous sample at 96 h, suggesting a possible role in the parasites’ response to nutrient deprivation (Fig. 4). When subjected to serum depletion, similar morphological changes were observed (Fig. 4e). Under this stress condition, the mRNA level of *Ehtbp* increased up to 231% compared to the control (Fig. 4f). This remarkable increase in the *Ehtbp* gene mRNA level led us to speculate that it behaves as a serum responsive factor during nutrient depletion by regulating an unknown group of genes on demand. We observed an increase in EhTBP protein level both in the nucleus and cytoplasm, which correlates with the upregulation of the mRNA level of *Ehtbp* under both stress conditions (Fig. 5 and Additional file 4: Figure S2). In contrast, *Homo sapiens tbp* (*Hstbp*) maintained a constitutive expression in serum starved quiescent cells, while an increase in the mRNA level of this gene was observed 15 min after serum stimulation [51]. In our study, *Ehtrf1* marked a constitutive expression throughout growth and serum depletion stress. However, TRF2 negatively regulates the cell cycle progression rate and is dispensable for the transition through the G2 phase in DT40 chicken cells [52]. A recent study on *E. histolytica* reports a long non-coding RNA (EhslncRNA), which responds to serum depletion at 24 h [53]. Our findings reveal that *Ehtbp* has an initial response at 12 h though the analysis of later time-points in future studies will be crucial to understanding the role of this gene in the *E. histolytica* biology.

Acute stress provokes in cells sudden changes in the transcriptional profile to respond to external stimuli [54]. In the case of trophozoites under heat-shock stress at 42 °C, we observed a negative regulation of *Ehtrf1*, which demands an in-depth study about its role in heat-shock stress. In the UV irradiation experiment, *Ehtbp* showed a basal mRNA level throughout the recovery time intervals. However, it might have a role in the UV-stress response, because TBP in other systems has more preference for binding damaged DNA induced by UV-irradiation than controlling transcriptional initiation [55]. The negative regulation of the mRNA level of *Ehtrf1* suggests that some responsive factors bind to *Ehtrf1* promoter responsive elements to provoke its decreased transcription, with the subsequent induction of genes involved in the UV damage response. This could be similar to the role of TRF2 in the induction of a transient change in a limited group of genes under UV-irradiation of DT40 cells [53]. We also determined the mRNA transcription levels of *Ehrad54, Ehblm, Ehpcna* genes which are involved in the response to UV-irradiation. The *Ehblm* gene displayed a similar expression pattern to that of *Ehtrf1*. However, this result is contrary to the increase in the amount of the Ehblm protein observed by others [39], which could be explained by an augment in the efficiency of *Ehblm* mRNA translation or an increase in the half-life of the protein through interactions with other proteins or due to post-translational modifications [56].

*Entamoeba histolytica* is well known for its virulence properties such as phagocytosis, host cell lysis and organ invasion for abscess formation. Erythrocytes are excellent target cells to study phagocytosis. Our results showed an engulfment capacity of 12 erythrocytes on average at 30 min. A slight decrease in the mRNA of *Ehtbp* was observed at 15 and 30 min. In the case of *Ehtrf1*, a well-noted decrease in the mRNA level was observed from 10 to 30 min due to unknown factors. This result led us to analyze their expression pattern in other virulence conditions. Analyzing the cytopathic effect is crucial for understanding the pathogenesis of this parasite during host cell invasion. In order to simulate the condition, we selected the MDCK cell line, the best model to study as a human epithelial barrier [57]. When live trophozoites were laid over the MDCK cell monolayer, the *Ehtbp* gene showed no change in its mRNA level whilst *Ehtrf1* had a transient increase at 5 min only.

Inside the host, abscesses first appear in the liver and dislodgement of virulent trophozoites occurs to acquire the other organs of the host [58]. Gilchrist et al. [59] showed in microarray analysis the negative regulation of *Ehtbp* both at days 1 and 29 during mice intestinal amoebic infection. However, trophozoites isolated from liver abscesses and cultured for 16 h showed a two-fold overexpression of *Ehtbp*, with no changes in *Ehtrf1* mRNA level [60]. In our case, trophozoites obtained from amoebic liver abscesses and cultured for 3–4 days, showed an unaltered *Ehtbp* mRNA level and downregulation of *Ehtrf1*. This result could be due to the shift from a solid tissue environment (hepatic liver) to TYI-S-33 medium.

Several approaches using siRNAs and ribozymes were intended to knockdown these transcription factors, but it was only successful with G3 trophozoites using the vector psAP2 for the *Ehtbp* gene [61, 62]. The main characteristics of the G3 strain are erythrophagocytosis and the incapability of cell monolayer destruction or abscess formation [62]. The significant decrease in the transcription level of *Ehtbp* silenced cells (Fig. 9a) greatly affected the rate of erythrophagocytosis (Fig. 9c). Moreover, we did not observe any growth defects (Additional file 5: Figure S3a) or morphological changes in trophozoites under this condition and in serum deprivation (data not shown). The reduction in the mRNA amount of *Ehtbp* might affect the expression of genes involved in erythrophagocytosis. However, the *EhrabB* and *Ehvps32* genes involved in phagocytosis were unaffected (Additional file 5: Figure S3b). The former has a GAAC motif at 22 to 29 bases upstream the ATG translation start site, but no TATA nor Inr boxes were identified [63]. The latter has a GAAC motif at -32 to -29 upstream the ATG codon site with no TATA and Inr consensus sequences. Two other genes (*Ehhgl* and *Ehrho*) containing the GAAC-box were evaluated by RT-qPCR, but only the *Ehhgl* diminished its mRNA level (Fig. 8d). This differential gene regulation suggests a specific subset of genes under the control of *Ehtbp* and *Ehtrf1*, which requires an extensive microarray analysis to comprehend and unveil the list of participating genes in phagocytosis or other physiological conditions.

For unknown reasons, the *Ehtrf1* gene was unable to get silenced in G3 trophozoites (data not shown). In addition, the use of specific antibodies against EhTBP and EhTRF1 to determine their protein expression levels has been elusive because both proteins are highly conserved. Even though we have eleven monoclonal antibodies for each protein, they recognized both polypeptides by Western blot (data not shown). This impedes the further use of antibodies for advanced techniques like chromatin immunoprecipitation experiments.

## Conclusions

EhTBP and EhTRF1 are two promiscuous transcription factors that displayed the DNA-binding capacity of diverse core promoter elements, including the GAAC-box, revealing them as putative GBPs. Remarkably, the differential expression observed under different stress stimuli and during the interaction with mammalian cells opens the possibility to evaluate key factors behind these phenomena. Targeting the signaling pathway during stringent conditions would unveil the transcriptional flexibility of these basal transcription factors and their selective response. Thus, elucidation of these basal transcription factors in response to those external insults will be useful to understanding the gene regulatory networks of this unicellular protozoan parasite. Hence, this study broadly opens the state of investigation, suggesting transcriptome analysis using either microarray analysis or next-generation sequencing to address the genes underregulation of these transcription factors in *E. histolytica*.

## Additional files

Additional file 1: Method for the calculation of dissociation constant values. (DOCX 20 kb)

Additional file 2: **Table S1.** Primers used for the amplification of selected genes. (DOCX 15 kb)

Additional file 3: **Figure. S1.** Quantification of DNA-protein complexes. (TIFF 552 kb)

Additional file 4: **Figure S2.** Immunolocalization of EhTBP in trophozoites during the growth culture (a) and serum depletion (b). (TIFF 371 kb)

Additional file 5: **Figure S3.** The *Ehtbp* knockdown does not affect the growth of G3 trophozoites. (TIFF 91 kb)

Additional file 6: **Table S2.** Coefficients of equations for *rEhTBP/DNA* probe. (DOCX 18 kb)

Additional file 7: **Table S3.** Coefficients of equations for *rEhTRF1/DNA* probe. (DOCX 21 kb)

## Abbreviations

ALA: Amebic liver abscess
EMSA: Electrophoretic mobility shift assay
GBP: GAAC-box binding protein
K_D_: Dissociation constant
MDCK: Madin-Darby canine kidney
NT: Normal trophozoites
TBP: TATA-box binding protein
TRF1: TBP-related factor 1
