# Supplementary material for: The Entamoeba histolytica TBP and TRF1 transcription factors are GAAC-box binding proteins, which display differential gene expression under different stress stimuli and during the interaction with mammalian cells
Source: Parasit Vectors. 2018 Mar 7;11:153. doi: 10.1186/s13071-018-2698-7 (PMC5842622; doi:10.1186/s13071-018-2698-7)
Supplement: Supplementary file 1 — Method for the calculation of dissociation constant values. (DOCX 20 kb) [file 13071_2018_2698_MOESM1_ESM.docx]

**Additional file 1**

**Method for the calculation of dissociation constant values**

The formation of complexes between recombinant proteins and radiolabeled double stranded oligonucleotide probes was measured as a function of protein concentration employing Eq. (1),

$F={(S_{x}-S_{0})}/{(S_{f}-S_{0})}$ (1)

where F is the fraction of DNA probe in DNA-protein complexes, *S_x_* is the amount of radioactivity in the shifted DNA-protein complex at protein concentration *x*, *S_0_* is the corresponding radioactivity at *x = 0*, and *S_f_* is the average amount of radioactivity when *F* becomes independent of *x*, i.e., when titration end point has been reached. For an accurate determination of *S_f_* values for each experiment, *S_x_* values were plotted for each *x* concentration of recombinant polypeptide tested. Then, the polynomial function that best describes the curve behavior was determined as follows:

$\ln S_{x}=a_{0}+ a_{1}x + a_{2}x^{2}+ a_{3}x^{3}+\cdots+a_{n}x^{n}+E-N(0,1)$ (2)

Where *ln S_x_* is the natural logarithm of *S_x_*, *a_n_* is the coefficient, *n* is the equation degree, and *E* is the residual error in the regression analysis [1]. The least square regression analysis was performed for fitness analysis. The degree of Eq. (2) determined in this work for both rEhTBP and rEhTRF1 and all DNA probes tested was *n = 2*. The statistical Student’s t-test (t) for each *a_n_* was estimated with the next equation:

$t_{n}={(a_{n}-0)}/{S_{a_{j}}}$ (3)

Where *j* has a value between *0* and *n*, and *Sa_n_* is the standard deviation of *a_n_* coefficient. The difference *(a_n_ − 0)* was considered as significant whether the Student’s t-test probability *P(t)* was lower than 0.05 [1].

After the calculation of coefficients of Eq. (2) for each experiment, the concentrations *x_max_* for rEhTBP or rEhTRF1 for all the probes tested corresponding to the maximum of curves, were determined by deriving Eq. (2) and solving it for zero value.

${d(\ln S_{x})}/{d\left( x \right)=0}$ (4)

Then values of *x_max_* for Eq. (2) were used to obtain the *S_f_* values and F was calculated with Eq. (1) for each *x* concentration of rEhTBP and rEhTRF1 used.

For the estimation of the molar ratios of total rEhTBP/DNA-probe and total rEhTRF1/DNA-probe when F = 1, the fractions of active rEhTBP and rEhTRF1 polypeptides binding to each of the DNA probes tested (active unbound plus active bound proteins) were determined assuming a binding stoichiometry of 1 [2]. Next, *F* was plotted as a function of total protein to DNA probe molar ratios. Following the procedure followed for Eq. (2), the *ln F* was fitted as a polynomial function of the molar ratios of total rEhTBP/DNA-probe or rEhTRF1/DNA-probe by the least square method. The maximum of these curves were determined as before. For an F value of 1 (i.e. the saturating point), the reciprocal of the *x* intercept was multiplied by the total concentration of rEhTBP or rEhTRF1 in order to get the total fraction of active rEhTBP or rEhTRF1 in reaction mixtures. This value was named *(x/DNA probe)_max_* and it corresponds to a value of F = 1 [2].

For determining the Confidence Intervals (CI) of predictions obtained for the polynomial functions, the value Yp ± CI was used, being Yp the predicted value by the function for an *x* value. CI is defined by Eq. (5)

$CI=\left[ 1/{t\left( 0.975, gl \right)} \right]\left\{ \left[ X^{'}R^{-1}\left. X \right] \right.\sigma^{2} \right\}^{1/2}$ (5)

Where t(0.975, *gl*) is the Student’s t-test for 0.975 percentile, and *gl* the degrees of freedom; *X’* is the vector of the values raised to the transposed *X* vector, *R^-1^* corresponds to the inverse of the regression matrix, and *σ^1/2^* is the residual variance.

For the calculation of dissociation constants *K_D_* of DNA-protein complexes, we used the Eq. (6) as described [1, 3].

$F=P/\left( K_{D}+P \right)$ (6)

Where *P* corresponds to the uncomplexed amount of active rEhTBP or rEhTRF1 polypeptides and is related to the total concentration (*P_T_*) of total active rEhTBP or rEhTRF1 by Eq. (7)

$P_{T}=P+PD$ (7)

PD is the concentration of rEhTBP or rEhTRF1 in the DNA-protein complexes. Since Eq. (6) is a hyperbolic function, then 1/F should fit to a linear function of the reciprocal of *P*, and the slope of this line corresponds to *K_D_*. Next, these two variables were fitted by means of a robust regression method [1, 3] that avoided the deleterious effect of data outliers on *K_D_* values. Calculation of coefficients and variances were performed by programming iterative algorithms that used least square estimates as initial values [1, 3]. The apparent association equilibrium constant *Ka* is defined as the reciprocal of *K_D_*.

**Dissociation constants of rEhTBP and rEhTRF1 for the different TATA variants**

The dissociation constants of rEhTBP and rEhTRF1 for the different TATA variants were determined as described [1, 3]. First, EMSA experiments were performed to quantify the amount of radioactivity (*S_x_*) in the DNA-protein complexes formed with increasing amounts of purified rEhTBP or rEhTRF1 polypeptides (Fig. S1, A to J). Then, the natural logarithms of *S_x_* values (*ln S_x_*) were plotted as a function of the different protein concentrations tested (Fig. S1, A′ to I′). Experimental points were fitted to a second-degree polynomial function that best describes the mathematical relationship of *ln S_x_* as a function of *x* (Fig. S1, A′ to I′; Tables S1 and S2). To determine the average amount of radioactivity *S_f_* in the DNA-protein complexes at the titration end point, Eq. (4) was used to calculate the *x* value (*x_max_*) that corresponds to the maximum of equation. Then, this *x_max_* value was substituted in Eq. (2) to determine the *S_f_* value. Next, *F* values were determined for each protein concentration used using Eq. (1) and the natural logarithms of *F* values were plotted as a function of the total protein/DNA probe molar ratios. These points were fitted to a second-degree polynomial function as well (Tables S1 and S2; see Fig. 2H and K as graph examples). The values of protein/DNA probe molar ratios were determined by deriving these equations to obtain the maximum of curves that correspond to an *F* value of 1. Finally, we obtained the reciprocal values of *F* to plot them as a function of the reciprocal values of concentration *P* (the uncomplexed amount of active rEhTBP or rEhTRF1 polypeptides), which were calculated with Eq. (7). The relationship of *F* and *P* is described by Eq. (6). The plots obtained were lineal (see Fig. 2, I and L as examples) and its slope corresponds to the *K_D_* value. The dissociation constants obtained for each TATA variant studied are shown in Table 1. The *K_D_* values of rEhTBP for all the TATA variants analyzed varied between (1.69±1.37) x 10^-12^ M and (3.98±0.16) x 10^-11^ M, while the *K_D_* values of rEhTRF1 for all TATA variants were between (3.98±1.96) x 10^-12^ M and (5.29±0.98) x 10^-11^ M. Remarkably, rEhTBP did not show DNA-binding affinity for the cAcTTAAA(9) variant.

**References**

1. de Dios-Bravo G, Luna-Arias JP, Riverón AM, Olivares-Trejo JJ, López-Camarillo C, Orozco E. *Entamoeba histolytica* TATA-box binding protein binds to different TATA variants in vitro. FEBS J. 2005;272:1354-1366.
2. Coleman RA, Pugh BF. Evidence for functional binding and stable sliding of the TATA binding protein on nonspecific DNA. J Biol Chem. 1995;270:13850–13859.
3. Castañon-Sanchez CA, Luna-Arias JP, de Dios-Bravo G, Herrera-Aguirre ME, Olivares-Trejo JJ, Orozco E, Hernandez JM. *Entamoeba histolytica*: A unicellular organism containing two active genes encoding for members of the TBP family. Protein Expr Purif. 2010;70:48-59.
