# Supplementary material for: The Entamoeba histolytica TBP and TRF1 transcription factors are GAAC-box binding proteins, which display differential gene expression under different stress stimuli and during the interaction with mammalian cells
Source: Parasit Vectors. 2018 Mar 7;11:153. doi: 10.1186/s13071-018-2698-7 (PMC5842622; doi:10.1186/s13071-018-2698-7)
Supplement: Supplementary file 2 — Table S1. Primers used for the amplification of selected genes. (DOCX 15 kb) [file 13071_2018_2698_MOESM2_ESM.docx]

**Table S1** List of primers used for the amplification of selected genes

| **Gene**  **(Accession ID)** | **Oligonucleotides** | **Tm °C** |
| --- | --- | --- |
| *Ehtbp* (EHI_020610) | Fwd 5’-TAGTAGGGTCATGTGATGTG  Rev 5’-CTTCATCTTTAGCACCTGTAAG | 60 |
| *Ehtrf1* (EHI_077240) | Fwd 5’-TATTTCCAGGTGTTGTATATCGT  Rev 5’-TTGTTCTATTTCTTTCCCTCCT | 60 |
| *Eh40Ss2* (EHI_020280) | Fwd 5’-ATTCGGAAATAGAAGAGGAGG  Rev 5’-ACCAAGCTTGGAAGATTAGT | 58 |
| *Ehrad54* (EHI_103840) | Fwd 5’-GACAAGGACCTAATGGAGAAC  Rev 5’-GTCTTAGCTCCAGAACATGAA | 60 |
| *Ehblm* (EHI_028890) | Fwd 5’-GAATGGTGTGATGGAGAGTTTA  Rev 5’-CTTCCTGCTCTTCCACTTT | 60 |
| *Ehpcna* (EHI_128450) | Fwd 5’-AGAACTTCAATCTGACGCTATT  Rev 5’-TTTCACACATAGCACCTTTAGT | 60 |
| *Ehhsp70* (EHI_197860) | Fwd 5’-GAATGAAAGTGATGATGAGAGGAG  Rev 5’-GTGAAATAACCAGGAACAGAAATAAC | 60 |
| *Ehvps32*  (EHI_169820) | Fwd 5’-CAGGAGCTGCTCAACATTTAAGA  Rev 5’-AGCTGCAGCAAAAGACCTGG | 60 |
| *Ehrab B*  (EHI_181240) | Fwd 5’-AATTGGAGATTCCGGTGTTG  Rev 5’-TTGACCTGCAGTATCCCAAA | 60 |
| *Ehtbp*-G3* | Fwd 5’-AGCTAGGCCTATGTCAACACCTGGAGATTTCT  Rev 5’-GCAT**GAGCTC**TCTTAATAATCTTTGCATACTTTTTCG | 60 |
| *Ehrho*  (EHI_190440) | Fwd 5’-TAAGTGGCAACCAGAAGTTAGA  Rev 5’-TTGAAGACCTTGGTCCGTAAT | 60 |
| *Ehhgl*  (EHI_012270) | Fwd 5’-ACAGTCGATGGAGTAACAATCT  Rev 5’-TCCTTGTCCTGCTGTGTATT | 60 |

**Stu* I sequence is underlined and *Sac* I sequence is in bold letters.
