# Supplementary figures and images for: The Entamoeba histolytica TBP and TRF1 transcription factors are GAAC-box binding proteins, which display differential gene expression under different stress stimuli and during the interaction with mammalian cells
Source: Parasit Vectors. 2018 Mar 7;11:153. doi: 10.1186/s13071-018-2698-7 (PMC5842622; doi:10.1186/s13071-018-2698-7)

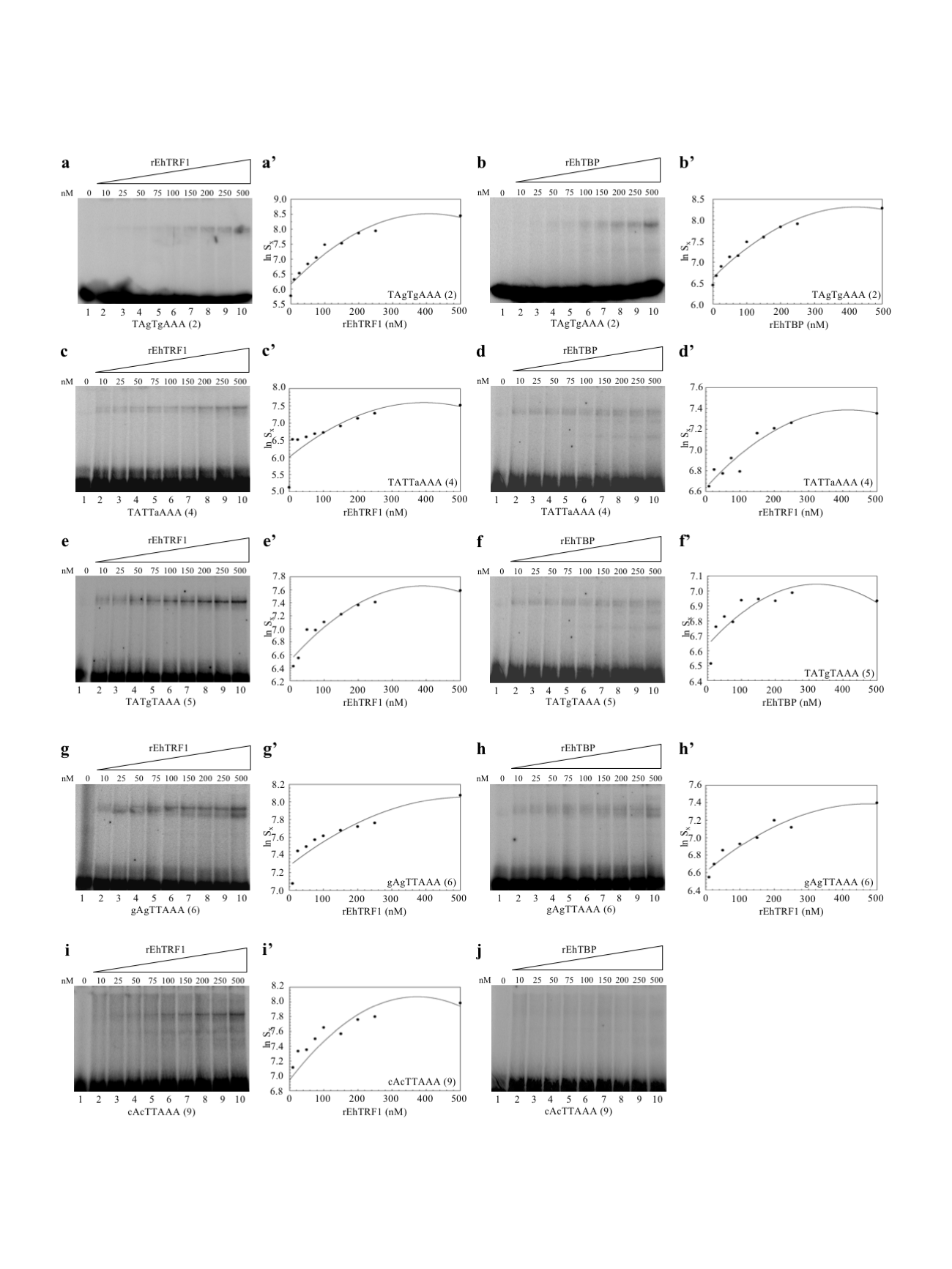

Supplement: Supplementary file 3 — Figure. S1. Quantification of DNA-protein complexes. (TIFF 552 kb) [file 13071_2018_2698_MOESM3_ESM.tiff]

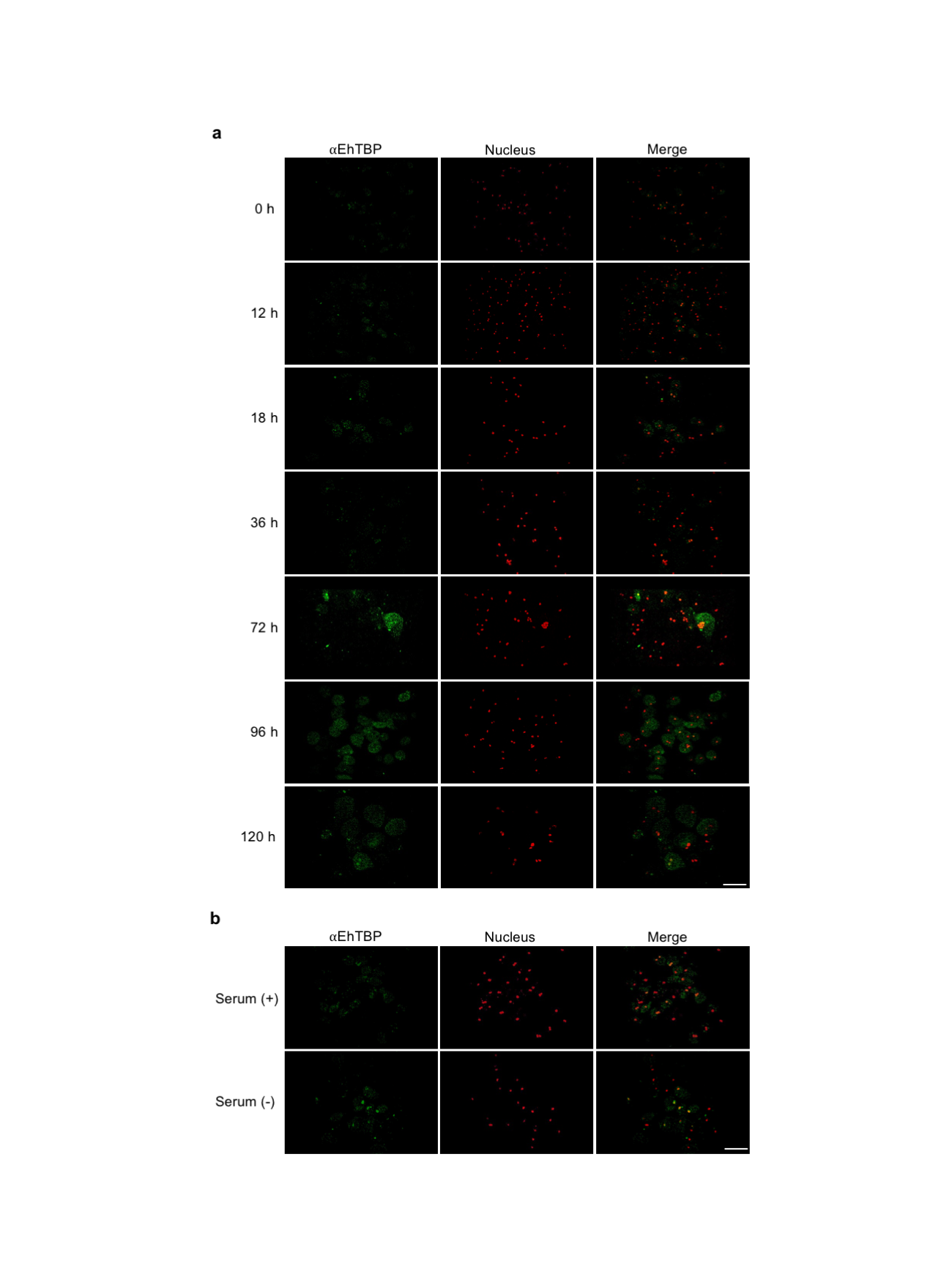

Supplement: Supplementary file 4 — Figure S2. Immunolocalization of EhTBP in trophozoites during the growth culture (a) and serum depletion (b). (TIFF 371 kb) [file 13071_2018_2698_MOESM4_ESM.tiff]

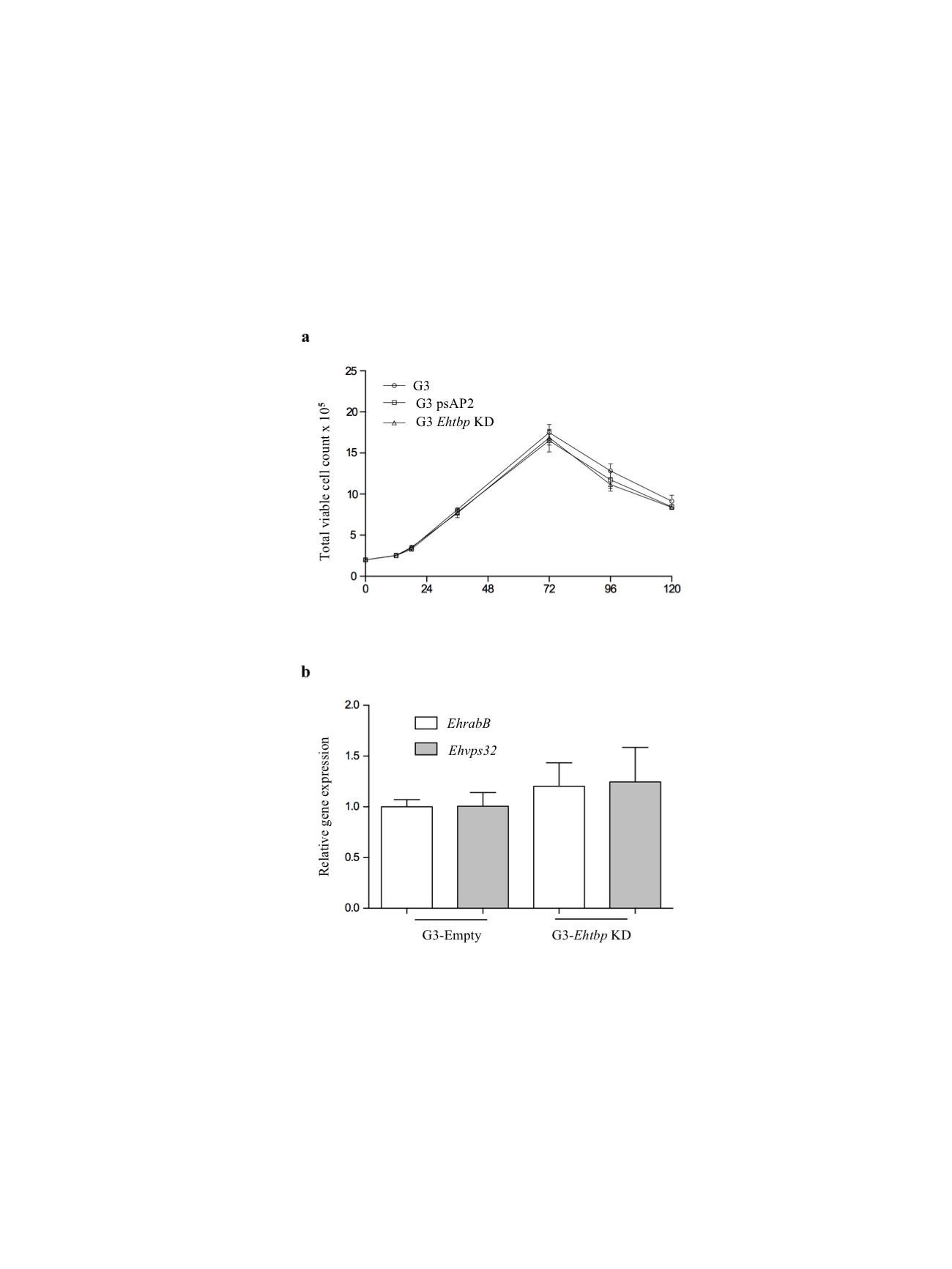

Supplement: Supplementary file 5 — Figure S3. The Ehtbp knockdown does not affect the growth of G3 trophozoites. (TIFF 91 kb) [file 13071_2018_2698_MOESM5_ESM.tiff]
