# Supplementary material for: The Entamoeba histolytica TBP and TRF1 transcription factors are GAAC-box binding proteins, which display differential gene expression under different stress stimuli and during the interaction with mammalian cells
Source: Parasit Vectors. 2018 Mar 7;11:153. doi: 10.1186/s13071-018-2698-7 (PMC5842622; doi:10.1186/s13071-018-2698-7)
Supplement: Supplementary file 6 — Table S2. Coefficients of equations for rEhTBP/DNA probe. (DOCX 18 kb) [file 13071_2018_2698_MOESM6_ESM.docx]

**Table S2** Coefficients of equations describing the mathematical relationships between *x* and *ln S_x_* for EhTBP, and *rEhTBP/DNA* probe molar ratio and *ln F*

| ***DNA sequence*** | ***a_0_*** | ***a_1_*** | ***a_2_*** | ***S^2^a_0_*** | ***S^2^a_1_*** | ***S^2^a_2_*** |
| --- | --- | --- | --- | --- | --- | --- |
| ***x* vs. *ln S_x_*** |  |  |  |  |  |  |
| TATTTAAA (1) | 7.0774 | 6.7×10^-3^ | -9.1210×10^-6^ | 0.1175 | 1.5×10^-3^ | 2.8876×10^-6^ |
| TAgTgAAA (2) | 6.6227 | 8.0×10^-3^ | -9.4474×10^-6^ | 0.0645 | 8.0×10^-4^ | 1.5849×10^-6^ |
| TATTggAA (3) | 6.3399 | 9.8×10^-3^ | -1.1934×10^-5^ | 0.0896 | 1.1×10^-3^ | 2.2029×10^-6^ |
| TATTaAAA (4) | 6.6368 | 3.6×10^-3^ | -4.3678×10^-6^ | 0.0580 | 7.0×10^-4^ | 1.2836×10^-6^ |
| TATgTAAA (5) | 6.6365 | 2.5×10^-3^ | -3.9491×10^-6^ | 0.0577 | 7.0×10^-4^ | 1.2769×10^-6^ |
| gAgTTAAA (6) | 6.6073 | 3.3×10^-3^ | -3.4427×10^-6^ | 0.0565 | 6.0×10^-4^ | 1.2066×10^-6^ |
| TAcTcAAA (7) | 8.2446 | 1.8×10^-3^ | -1.1780×10^-6^ | 0.0246 | 3.0×10^-4^ | 5.4554×10^-7^ |
| cAcTcAAA (8) | 6.5853 | 1.1×10^-2^ | -1.6711×10^-5^ | 0.1374 | 1.7×10^-3^ | 3.3768×10^-6^ |
| cAcTTAAA (9) |  |  |  |  |  |  |
| TATTTttt (10) | 6.2928 | 4.0×10^-3^ | -5.4505×10^-6^ | 0.1286 | 1.6×10^-3^ | 3.1608×10^-6^ |
| GAAC-box | 6.5627 | 8.3×10^-3^ | -1.3159×10^-5^ | 0.1161 | 1.4×10^-3^ | 2.8540×10^-6^ |
| ***rEhTBP/DNA probe molar ratio* vs *lnF*** |  |  |  |  |  |  |
| TATTTAAA (1) | -1.5566 | 6.0×10^-4^ | -6.7565×10^-8^ | 0.0650 | 5.8×10^-5^ | 1.0411×10^-8^ |
| TAgTgAAA (2) | -3.5919 | 1.5×10^-3^ | -1.5139×10^-7^ | 0.1678 | 2.0×10^-4^ | 3.0199×10^-8^ |
| TATTggAA (3) | -3.7901 | 1.3×10^-3^ | -1.0327×10^-7^ | 0.3450 | 3.0×10^-4^ | 5.8110×10^-8^ |
| TATTaAAA (4) | -1.0290 | 2.0×10^-4^ | -1.0615×10^-8^ | 0.0694 | 3.0×10^-5^ | 2.4650×10^-9^ |
| TATgTAAA (5) | -0.4691 | 8.7241×10^-5^ | -3.8427×10^-9^ | 0.0725 | 2.4×10^-5^ | 1.2601×10^-9^ |
| gAgTTAAA (6) | -1.1525 | 2.0×10^-4^ | -8.2426×10^-9^ | 0.1301 | 5.8×10^-5^ | 4.9337×10^-9^ |
| TAcTcAAA (7) | -2.6175 | 3.0×10^-4^ | 5.9652×10^-9^ | 0.0407 | 7.9×10^-5^ | 2.9806×10^-8^ |
| cAcTcAAA (8) | -3.1100 | 1.6×10^-3^ | -2.0220×10^-7^ | 0.2834 | 2.0×10^-4^ | 3.5620×10^-8^ |
| cAcTTAAA (9) |  |  |  |  |  |  |
| TATTTttt (10) | -0.8920 | 9.4512×10^-5^ | -2.8689×10^-9^ | 0.1222 | 3.8×10^-5^ | 2.1457×10^-9^ |
| GAAC-box | -2.0250 | 8.0×10^-4^ | -6.4954×10^-8^ | 0.1844 | 1.0×10^-4^ | 1.1236×10^-8^ |

*a_0_,* *a_1_* and *a_2_* are the coefficients of equations *ln Sx = a_2_x^2^ + a_1_x + a_0_ + E - N(0, 1)*, and *ln F = a_2_(rEhTBP/DNA probe)^2^ + a_1_(rEhTBP/DNA probe) + a_0_ + E - N(0, 1)* and *ln F = a_2_(rEhTBP/DNA probe)^2^ + a_1_(rEhTBP/DNA probe) + a_0_ + E - N(0, 1)*. *Sa_i_* is the standard deviation of *a_i_* coefficients.
