# Supplementary material for: The Entamoeba histolytica TBP and TRF1 transcription factors are GAAC-box binding proteins, which display differential gene expression under different stress stimuli and during the interaction with mammalian cells
Source: Parasit Vectors. 2018 Mar 7;11:153. doi: 10.1186/s13071-018-2698-7 (PMC5842622; doi:10.1186/s13071-018-2698-7)
Supplement: Supplementary file 7 — Table S3. Coefficients of equations for rEhTRF1/DNA probe. (DOCX 21 kb) [file 13071_2018_2698_MOESM7_ESM.docx]

**Table S3** Coefficients of equations describing the mathematical relationships between *x* and *ln S_x_* for EhTRF1, and *rEhTRF1/DNA* probe molar ratio and *ln F*

| ***DNA sequence*** | ***a_0_*** | ***a_1_*** | ***a_2_*** | ***S^2^a_0_*** | ***S^2^a_1_*** | ***S^2^a_2_*** |
| --- | --- | --- | --- | --- | --- | --- |
| ***x* vs. *ln S_x_*** |  |  |  |  |  |  |
| TATTTAAA (1) | 6.4745 | 1.2×10^-2^ | -1.6170×10^-5^ | 0.1753 | 2.2×10^-3^ | 4.3092×10^-6^  3.0413×10^-6^  7.0837×10^-6^  5.5695×10^-6^  1.8833×10^-6^  1.8228×10^-6^  8.0214×10^-7^  1.5559×10^-6^ |
| TAgTgAAA (2) | 6.1586 | 1.2×10^-2^ | -1.4259×10^-5^ | 0.1237 | 1.5×10^-3^ |  |
| TATTggAA (3) | 5.6929 | 1.6×10^-2^ | -2.1777×10^-5^ | 0.2881 | 3.6×10^-3^ |  |
| TATTaAAA (4) | 6.0064 | 8.1×10^-3^ | -1.0265×10^-5^ | 0.2266 | 2.8×10^-3^ |  |
| TATgTAAA (5) | 6.5077 | 5.9×10^-3^ | -7.6102×10^-6^ | 0.0850 | 1.0×10^-3^ |  |
| gAgTTAAA (6) | 7.7258 | 3.0×10^-3^ | -2.9107×10^-6^ | 0.0823 | 1.0×10^-3^ |  |
| TAcTcAAA (7)  cAcTcAAA (8) | 7.9733 | 4.3×10^-3^ | -4.6130×10^-6^ | 0.0362 | 4.0×10^-3^ |  |
|  | 6.9077 | 6.9×10^-3^ | -9.0244×10^-6^ | 0.0633 | 8.0×10^-4^ |  |
| cAcTTAAA (9) | 7.1925 | 3.7×10^-3^ | -4.1976×10^-6^ | 0.0596 | 7.0×10^-4^ | 1.3197×10^-6^ |
| TATTTttt (10)  GAAC-box | 6.1324 | 9.1×10^-3^ | -1.1787×10^-5^ | 0.1713 | 2.1×10^-3^ | 4.2117×10^-6^ |
|  | 6.5370 | 1.5×10^-2^ | -2.1816×10^-5^ | 0.2181 | 2.7×10^-3^ | 5.3617×10^-6^ |
| ***rEhTRF1/DNA probe molar ratio* vs *lnF*** |  |  |  |  |  |  |
| TATTTAAA (1) | -3.5274 | 1.9×10^-3^ | -2.7377×10^-7^ | 0.2667 | 3.0×10^-4^ | 6.5526×10^-8^ |
| TAgTgAAA (2) | -3.7381 | 1.0×10^-3^ | -2.6928×10^-8^ | 0.5441 | 5.0×10^-4^ | 1.0909×10^-7^ |
| TATTggAA (3) | -3.4524 | 9.0×10^-4^ | -1.8119×10^-8^ | 2.8150 | 2.1×10^-3^ | 3.7136×10^-7^ |
| TATTaAAA (4) | -1.4494 | 1.0×10^-4^ | 1.7845×10^-9^ | 0.1351 | 6.0×10^-5^ | 5.7535×10^-9^ |
| TATgTAAA (5) | -1.4549 | 4.0×10^-4^ | -2.2159×10^-8^ | 0.1101 | 5.6×10^-5^ | 5.6357×10^-9^ |
| gAgTTAAA (6) | -0.0827 | 3.0×10^-4^ | -1.9492×10^-8^ | 0.1324 | 1.0×10^-4^ | 1.9550×10^-8^  9.5196×10^-8^  3.3564×10^-8^  1.5235×10^-8^  3.1026×10^-8^  7.9528×10^-8^ |
| TAcTcAAA (7) | -1.4646 | 1.1×10^-3^ | -1.6725×10^-7^ | 0.1139 | 2.0×10^-4^ |  |
| cAcTcAAA (8) | -2.8563 | 1.3×10^-3^ | -1.4832×10^-7^ | 0.2223 | 2.0×10^-4^ |  |
| cAcTTAAA (9) | -1.3009 | 4.0×10^-4^ | -3.5930×10^-8^ | 0.1235 | 9.8×10^-5^ |  |
| TATTTttt (10) | -2.4003 | 6.0×10^-4^ | -5.0547×10^-8^ | 0.4366 | 3.0×10^-4^ |  |
| GAAC-box | -4.0235 | 3.8×10^-4^ | -9.0373×10^-7^ | 0.1807 | 3.0×10^-4^ |  |

*a_0_,* *a_1_* and *a_2_* are the coefficients of equations *ln Sx = a_2_x^2^ + a_1_x + a_0_ + E - N(0, 1)*, and *ln F = a_2_(rEhTRF1/DNA probe)^2^ + a_1_(rEhTRF1/DNA probe) + a_0_ + E - N(0, 1)* and *ln F = a_2_(rEhTRF1/DNA probe)^2^ + a_1_(rEhTRF1/DNA probe) + a_0_ + E - N(0, 1)*. *Sa_i_* is the standard deviation of *a_i_* coefficients.
